# Supplementary figures and images for: Streptococcus pneumoniae synchronizes the states of cell wall peptidoglycan acetylation and genome methylation by programmed DNA inversions
Source: PLoS Pathog. 2025 Aug 5;21(8):e1013286. doi: 10.1371/journal.ppat.1013286 (PMC12324116; doi:10.1371/journal.ppat.1013286)

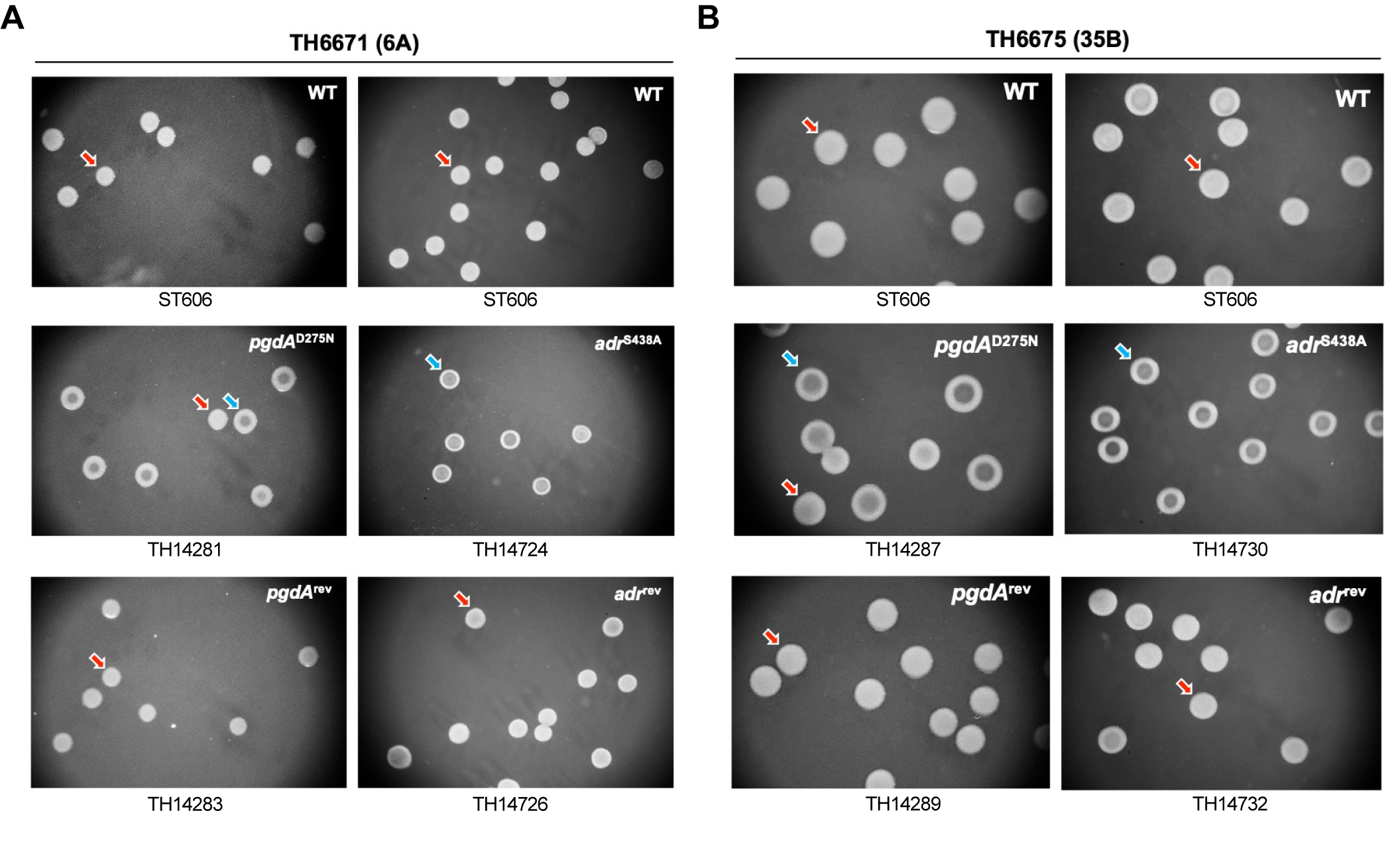

Supplement: S1 Fig — Red and blue arrowheads indicate the representative opaque (O) and transparent (T) colonies, respectively. (TIF) [file ppat.1013286.s001.tif]

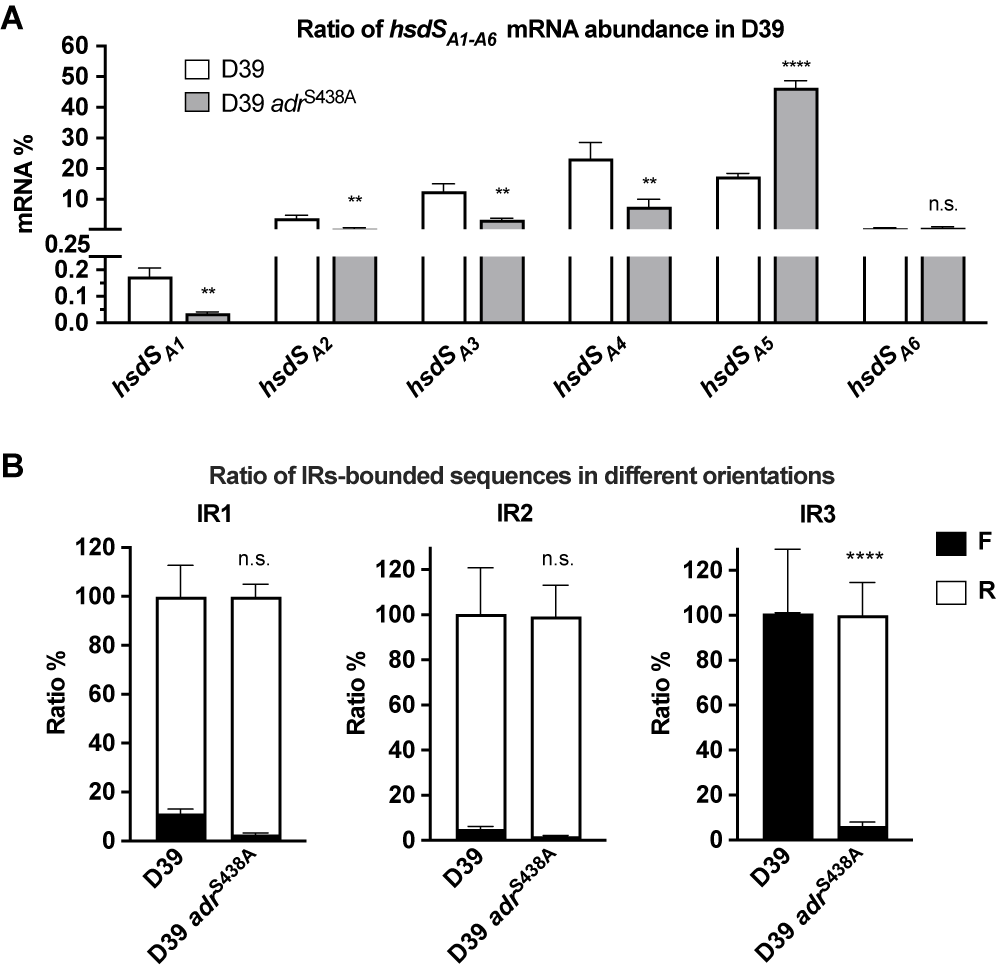

Supplement: S2 Fig — (A) The proportions of six hsdSA allelic variants in single populations of strain D39 or its adrS438A derivative were assessed by qRT-PCR using allele-specific primer sets. Data shown as mean ± s.d. of 3 replicates in a representative experiment. (B) The ratio of IR1-, IR2-, and IR3-bound sequences in different orientations in strain D39 or its adrS438A derivative are shown as in Fig 2E. (TIF) [file ppat.1013286.s002.tif]

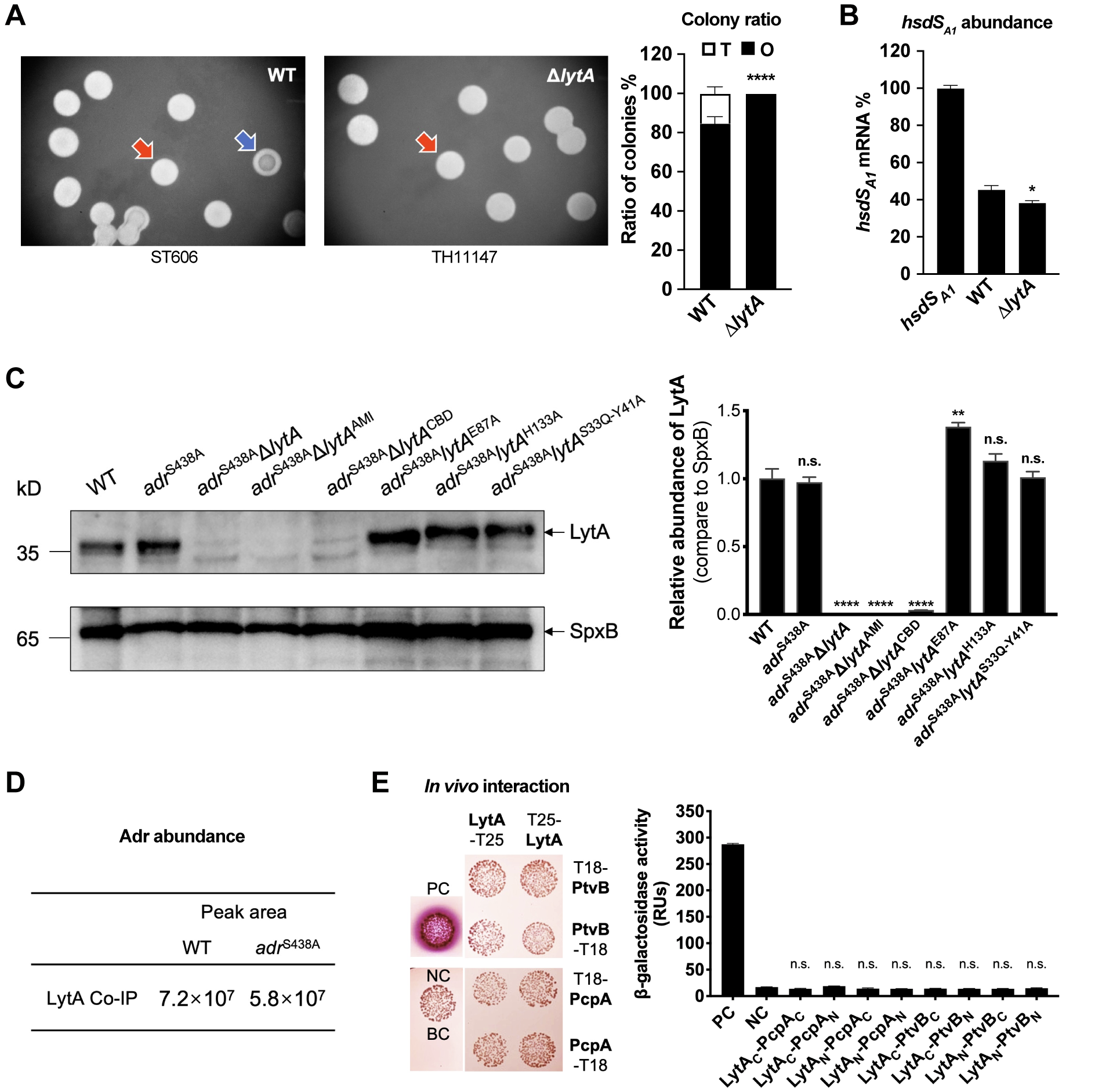

Supplement: S3 Fig — (A to B) The colony phenotypes (A) and relative abundance of the hsdSA1 mRNA (B) of lytA mutant. (C) The abundance of LytA in ST606 (WT) derivatives. LytA was assessed by Western blotting using a rabbit antiserum (left panel). The relative protein abundance of LytA was calculated by normalization to the band density of the internal control pyruvate oxidase SpxB (right panel). (D) The Adr abundance in the whole protein lysates of ST606 and adrS438A strains is presented as the average of the peak area obtained from two biological repeats in a representative experiments. (E) Detection of interactions between LytA and its associated proteins by bacterial two-hybrid assay. The β-galactosidase activity is assessed and presented for each reporter strain. PC, positive control (pKT25-zip and pUT18C-zip), NC, negative control (empty vectors pKT25 and pUT18C). Significance between NC and experimental groups is presented. (TIF) [file ppat.1013286.s003.tif]

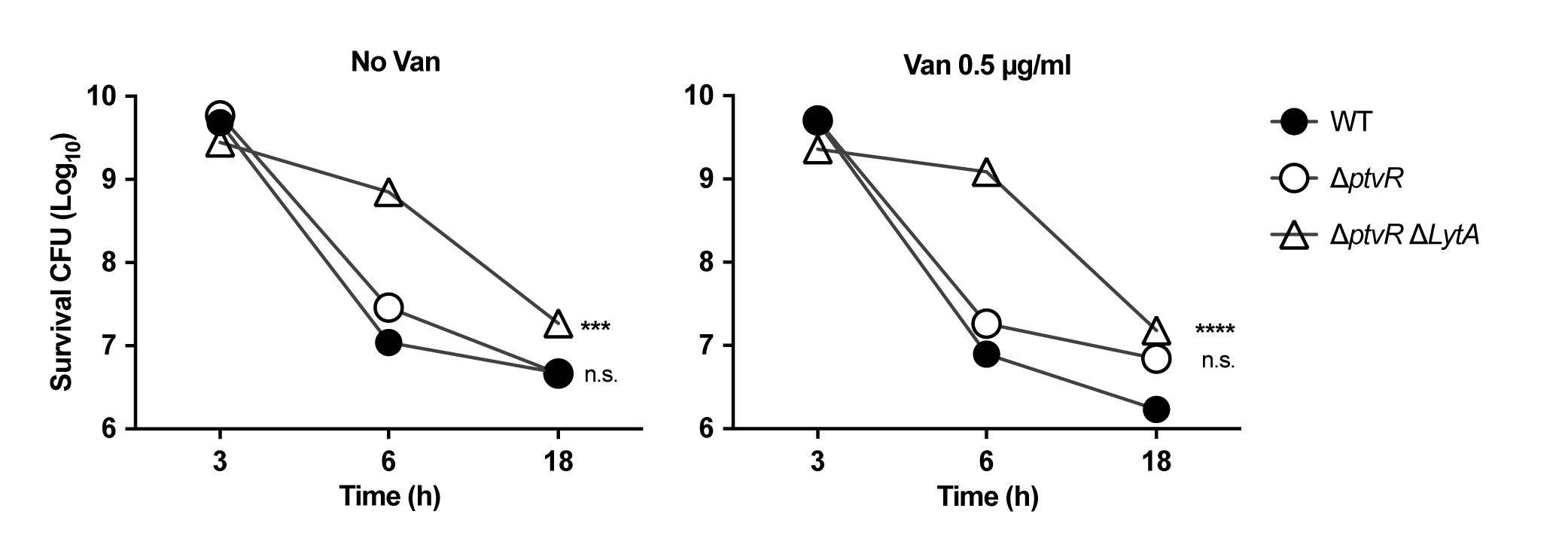

Supplement: S4 Fig — Pneumococci were cultured to an OD620 of 0.5 in THY medium before being incubated in the presence or absence of 0.5 μg/ml vancomycin under routine pneumococcal culture conditions. Bacterial survival was assessed by plating for CFU at various time points. (TIF) [file ppat.1013286.s004.tif]

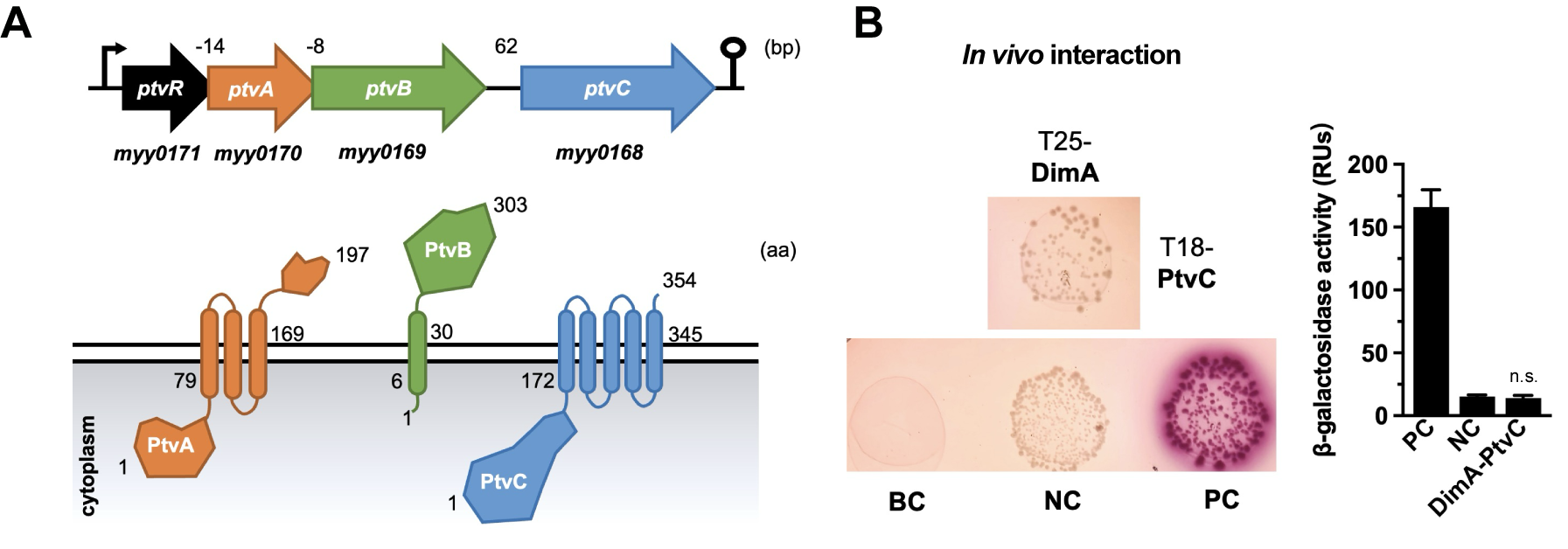

Supplement: S5 Fig — (A) The genetic (upper panel) and protein (lower panel) features of the ptv locus. The ptvR gene encodes a negative regulator of this operon. The nucleotides between two adjacent genes are marked in base pairs (bp). The promoter and rho-independent transcription terminator are indicated by a black arrow and a hairpin. Lower panel depicts the predicted protein structure of PtvA, PtvB, and PtvC. The number of the amino acid (aa) at various regions are indicated. The transmembrane topology was predicted using TMHMM - 2.0 tool. (B) Detection of interactions between PtvC and DimA by bacterial two-hybrid assay. Colonies on the MacConkey/maltose plates (left panel) and β-galactosidase activity (right panel) are shown for each reporter strain. PC, positive control (pKT25-zip and pUT18C-zip), NC, negative control (empty vectors pKT25 and pUT18C). BC, blank control without plasmid. Significance between NC and the experimental group is presented. (TIF) [file ppat.1013286.s005.tif]

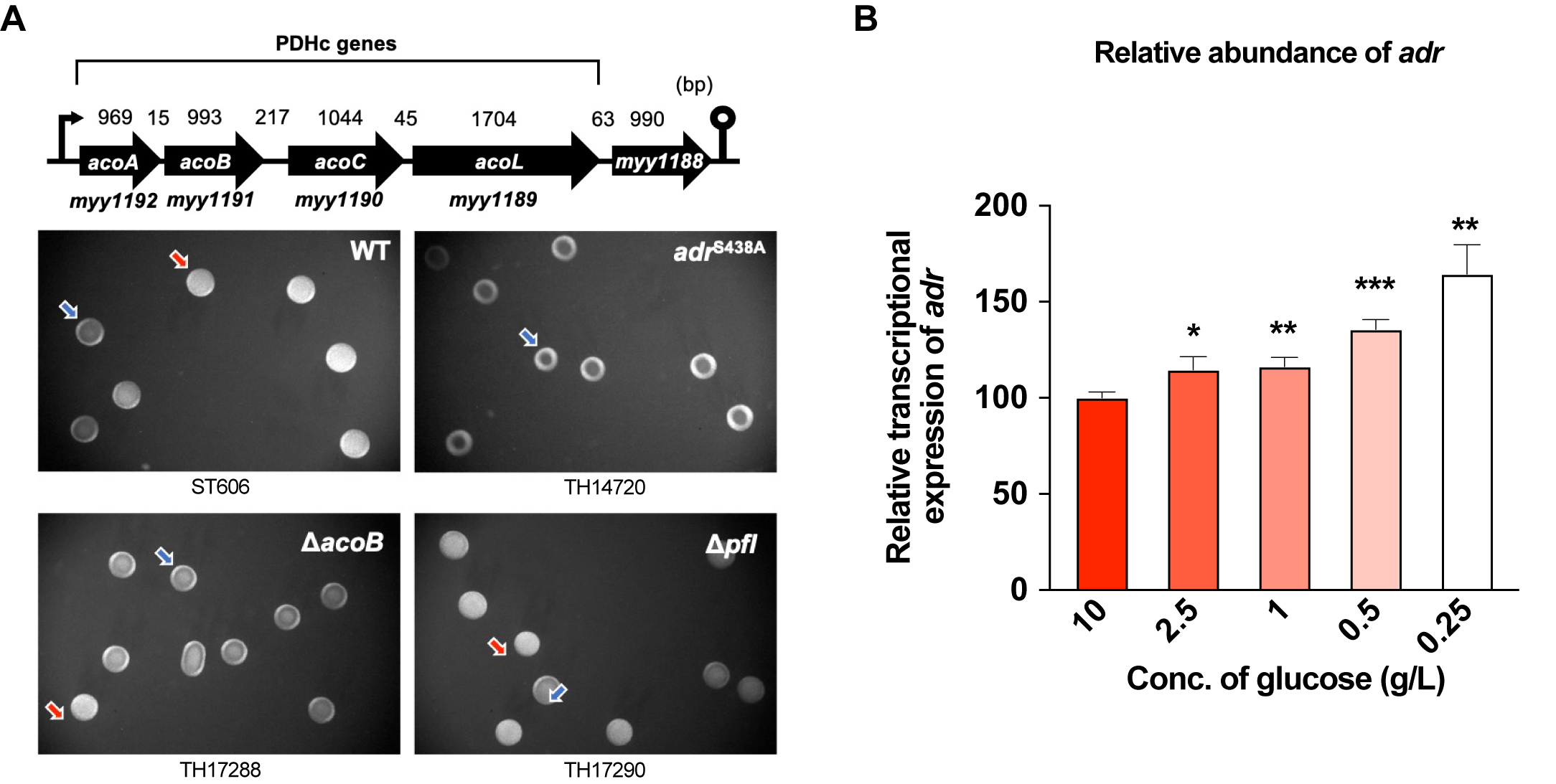

Supplement: S6 Fig — (A) Representative colonies of acoB and pfl mutants. Top panel indicates the organization of genes encoding PDHC. Colonies indicated by red and blue arrowheads represent O and T colonies, respectively. (B) The transcription of adr in pneumococci cultured in CDM with different concentrations of glucose. The mRNA of adr was detected by qRT-PCR and normalized to that of the internal control era. (TIF) [file ppat.1013286.s006.tif]
