# Supplementary material for: Streptococcus pneumoniae synchronizes the states of cell wall peptidoglycan acetylation and genome methylation by programmed DNA inversions
Source: PLoS Pathog. 2025 Aug 5;21(8):e1013286. doi: 10.1371/journal.ppat.1013286 (PMC12324116; doi:10.1371/journal.ppat.1013286)
Supplement: S1 Table — (DOCX) [file ppat.1013286.s007.docx]

**S1 Table. Methylation sequences specified by the Spn556I/III MTases^a^**

| **Genotype** | **Spn556I MTase** | | | **Spn556III MTases HsdS_1_** | | |
| --- | --- | --- | --- | --- | --- | --- |
|  | **5’-TCTAG^m6^A-3’**  **3’-^m6^AGATCT-5’** | | | **5’-GAT^m6^AN_7_TCA-3’**  **3’-CTATN_7_^m6^AGT-5’** | | |
|  | # in  genome^b^ | # detected^c^ | %  detected^d^ | # in genome | # detected | % detected |
| WT | 664 | 664 | 100 | 1246 | 1246 | 100 |
| *adr*^S438A^ | 664 | 664 | 100 | 1246 | 1246 | 100 |
| *pgdA*^D275N^ | 664 | 565 | 94.2 | 1246 | 979 | 78.6 |

^a^The accumulative number of all methylated loci in each strain exceeded 100% because a base was considered as being methylated once more than 30% of all the reads at the position passed the cutoff value in the PacBio platform.

^b^Total number of loci in both DNA strands in the genome of ST556 (accession CP003357.2).

^c^Total loci detected by the SMRT sequencing.

^d^Percentage of the detected motifs was calculated as follows: total loci detected/total loci in the genome.

N, any nucleotide.
