# Supplementary material for: Streptococcus pneumoniae synchronizes the states of cell wall peptidoglycan acetylation and genome methylation by programmed DNA inversions
Source: PLoS Pathog. 2025 Aug 5;21(8):e1013286. doi: 10.1371/journal.ppat.1013286 (PMC12324116; doi:10.1371/journal.ppat.1013286)
Supplement: S2 Table — (DOCX) [file ppat.1013286.s008.docx]

**S2 Table. SMRT sequencing data of *pgdA* and *adr* mutants**

| **Strain** | **Mean concordance** | **Number of reads** | **Number of bases (bp)** | **Mean read length (bp)** | **Median read length (bp)** |
| --- | --- | --- | --- | --- | --- |
| WT | 0.88 | 53,057 | 623,719,683 | 11,756 | 13,714 |
| *pgdA*^D275N^ | 0.88 | 74,677 | 549,708,649 | 7,361 | 11,297 |
| *adr*^S438A^ | 0.88 | 57,769 | 623,127,995 | 10,787 | 12,973 |
