# Supplementary material for: Streptococcus pneumoniae synchronizes the states of cell wall peptidoglycan acetylation and genome methylation by programmed DNA inversions
Source: PLoS Pathog. 2025 Aug 5;21(8):e1013286. doi: 10.1371/journal.ppat.1013286 (PMC12324116; doi:10.1371/journal.ppat.1013286)
Supplement: S3 Table — (DOCX) [file ppat.1013286.s009.docx]

**S3 Table. LytA-associated proteins changed in the Adr-inactivated mutant ^a^**

| **Gene ID** | **Description** | **MW ^b^ (kDa)** | **Abundance in**  **LytA Co-IP ^c^** | | |  | **Abundance in**  **whole-cell lysate ^c^** | | |
| --- | --- | --- | --- | --- | --- | --- | --- | --- | --- |
|  |  |  | **WT** | ***adr*^S438A^** | **Fold change** |  | **WT** | ***adr*^S438A^** | **Fold change** |
| MYY2056 | Pneumococcal choline-binding protein, PcpA | 73.84 | 1.09E7 | 0 | -∞ |  | 4.99E7 | 6.70E7 | 1.34 |
| MYY0960 | DNA-binding protein, YceD | 20.37 | 6.05E6 | 0 | -∞ |  | 3.02E7 | 5.75E7 | 1.90 |
| MYY0111 | Phosphate acyltransferase, PlsX | 34.90 | 1.41E7 | 2.24E6 | -6.29 |  | 2.51E8 | 4.24E8 | 1.69 |
| MYY1573 | Manganese ABC transporter ATP-binding protein, ZnuC | 26.86 | 1.74E7 | 3.32E6 | -5.24 |  | 1.16E8 | 2.76E8 | 2.37 |
| MYY1663 | Ribosomal silencing factor, RsfS | 12.85 | 3.86E7 | 8.30E6 | -4.65 |  | 2.77E7 | 1.55E8 | 5.60 |
| MYY0791 | Cell division protein, FtsX | 34.31 | 1.25E7 | 3.30E6 | -3.79 |  | 2.22E8 | 3.02E8 | 1.36 |
| MYY1559 | Threonyl-tRNA synthase, ThrS | 74.67 | 1.12E8 | 3.21E7 | -3.48 |  | 3.76E8 | 3.43E8 | -1.10 |
| MYY0169 | Phenotypic tolerance to vancomycin, PtvB | 34.03 | 2.68E7 | 8.53E6 | -3.14 |  | 7.96E7 | 1.54E8 | 1.94 |
| MYY03060 | Hypothetical protein | 27.86 | 9.14E6 | 2.97E6 | -3.08 |  | 2.24E8 | 3.53E8 | 1.57 |
| MYY0914 | Chorismate synthase | 42.85 | 2.12E7 | 7.40E6 | -2.86 |  | 2.12E8 | 2.11E8 | -1.01 |
| MYY1707 | General stress protein | 21.80 | 5.34E7 | 1.88E7 | -2.85 |  | 1.08E9 | 1.93E9 | 1.79 |
| MYY0806 | 30S ribosomal protein S16, RpsP | 10.22 | 4.43E8 | 1.84E8 | -2.40 |  | 1.24E9 | 1.59E9 | 1.28 |
| MYY1153 | 50S ribosomal protein L21, RplU | 11.19 | 3.15E8 | 1.33E8 | -2.38 |  | 2.09E9 | 1.05E9 | -1.98 |
| MYY0726 | GTP-binding protein, TypA | 68.14 | 7.92E7 | 3.36E7 | -2.36 |  | 7.35E8 | 7.10E8 | -1.04 |
| MYY1352 | Heme ABC transporter ATP-binding protein, YufO | 55.10 | 5.38E7 | 2.46E7 | -2.19 |  | 4.27E8 | 4.68E8 | 1.10 |
| MYY0817 | Glycyl-tRNA ligase | 34.91 | 6.92E7 | 3.32E7 | -2.08 |  | 7.60E8 | 1.18E9 | 1.55 |

**^a^** The abundance of proteins pulled down by LytA was detected by LC-MS/MS. Candidate selection standard: |fold change| ≥ 2, score ≥ 5.

**^b^** MW, the molecular weight of the protein in strain ST556.

**^c^** The abundance of each protein was shown as the average of the peak area obtained from 4 repeats in 2 individual experiments.

Gray background indicates the proteins essential for pneumococcal viability (encoded by essential genes).
