# Supplementary material for: Streptococcus pneumoniae synchronizes the states of cell wall peptidoglycan acetylation and genome methylation by programmed DNA inversions
Source: PLoS Pathog. 2025 Aug 5;21(8):e1013286. doi: 10.1371/journal.ppat.1013286 (PMC12324116; doi:10.1371/journal.ppat.1013286)
Supplement: S4 Table — (DOCX) [file ppat.1013286.s010.docx]

**S4 Table. PtvB-associated proteins changed in the Adr-inactivated mutant ^a^**

|  | **Gene ID** | | **Description** | **MW ^b^**  **(kDa)** | **Abundance in**  **PtvB Co-IP ^c^** | | |  | **Abundance in**  **whole-cell lysate ^c^** | | |
| --- | --- | --- | --- | --- | --- | --- | --- | --- | --- | --- | --- |
|  |  |  |  |  | ***adr*^S438A^** | **WT** | **Fold change** |  | ***adr*^S438A^** | **WT** | **Fold change** |
| **Significantly increased in *adr*^S438A^** | | | | | | | | | | | |
|  | | MYY0606 | tRNA (guanine-N7)-methyltransferase | 24.3 | 8.16E6 | 0 | +∞ |  | 1.21E8 | 6.07E7 | 2.00 |
|  | | MYY0439 | dTDP-glucose 4,6-dehydratase | 39.0 | 4.98E7 | 8.66E6 | 5.75 |  | 1.02E9 | 6.76E8 | 1.50 |
|  | | MYY0497 | Acetyl-CoA carboxylase subunit alpha | 28.2 | 4.74E8 | 1.15E8 | 4.12 |  | 5.93E8 | 4.33E8 | 1.37 |
|  | | MYY1451 | UDP-N-acetylmuramate--alanine ligase | 49.8 | 9.04E6 | 2.38E6 | 3.79 |  | 5.76E8 | 3.71E8 | 1.55 |
|  | | MYY0734 | Orotidine 5'-phosphate decarboxylase | 25.4 | 6.79E6 | 1.84E6 | 3.69 |  | 2.36E8 | 1.36E8 | 1.74 |
|  | | MYY2127 | 50S ribosomal protein L9 | 16.5 | 1.32E8 | 3.74E7 | 3.52 |  | 5.99E8 | 5.54E8 | 1.08 |
|  | | MYY1025 | Serine/threonine-protein kinase MRCK beta (DimA) | 49.1 | 1.45E7 | 4.55E6 | 3.19 |  | 3.51E7 | 4.14E7 | 0.85 |
|  | | MYY0496 | acetyl-CoA carboxylase subunit beta | 31.8 | 8.42E8 | 2.70E8 | 3.12 |  | 4.08E8 | 2.82E8 | 1.44 |
|  | | MYY0914 | Chorismate synthase | 42.8 | 3.48E7 | 1.14E7 | 3.05 |  | 2.11E8 | 2.12E8 | 0.99 |
|  | | MYY0624 | Valine--tRNA ligase | 100.8 | 2.13E8 | 7.10E7 | 3.00 |  | 2.95E8 | 3.70E8 | 0.80 |
|  | | MYY1470 | Single-stranded DNA-binding protein | 17.3 | 1.51E8 | 5.08E7 | 2.98 |  | 9.25E8 | 5.40E8 | 1.71 |
|  | | MYY0571 | Restriction endonuclease subunit M | 56.4 | 5.76E6 | 1.94E6 | 2.96 |  | 1.01E8 | 8.59E7 | 1.18 |
|  | | MYY0735 | Orotate phosphoribosyltransferase | 22.8 | 5.72E7 | 2.05E7 | 2.80 |  | 9.48E8 | 5.79E8 | 1.64 |
|  | | MYY0303 | 50S ribosomal protein L5 | 19.8 | 8.42E9 | 3.16E9 | 2.66 |  | 7.21E10 | 4.74E10 | 1.52 |
|  | | MYY1974 | Alcohol dehydrogenase | 38.1 | 8.31E7 | 3.19E7 | 2.60 |  | 2.65E9 | 8.76E8 | 3.03 |
|  | | MYY0806 | 30S ribosomal protein S16 | 10.2 | 1.14E8 | 4.42E7 | 2.58 |  | 1.59E9 | 1.24E9 | 1.28 |
|  | | MYY1580 | Phosphoglycerate mutase | 26.0 | 1.43E8 | 5.60E7 | 2.55 |  | 5.93E9 | 4.23E9 | 1.40 |
|  | | MYY0002 | DNA polymerase III subunit beta | 42.0 | 2.68E7 | 1.06E7 | 2.54 |  | 8.69E8 | 5.26E8 | 1.65 |
|  | | MYY0128 | Phosphoribosyl carboxyaminoimidazole mutase | 16.9 | 5.51E7 | 2.20E7 | 2.50 |  | 3.62E8 | 3.17E8 | 1.14 |
|  | | MYY2032 | Aspartate--tRNA ligase | 66.2 | 3.92E7 | 1.59E7 | 2.46 |  | 4.17E8 | 3.70E8 | 1.13 |
|  | | MYY1988 | Glutamyl-tRNA synthetase | 55.9 | 4.42E7 | 1.81E7 | 2.44 |  | 3.88E8 | 3.54E8 | 1.10 |
|  | | MYY0041 | Hypoxanthine phosphoribosyltransferase | 20.1 | 2.31E7 | 9.96E6 | 2.32 |  | 6.67E8 | 5.04E8 | 1.32 |
|  | | MYY1493 | Phosphoglucosamine mutase | 48.1 | 3.63E7 | 1.58E7 | 2.30 |  | 6.40E8 | 6.01E8 | 1.06 |
|  | | MYY1537 | UDP-galactose-4-epimerase | 37.4 | 3.87E7 | 1.71E7 | 2.27 |  | 2.71E9 | 1.75E9 | 1.55 |
|  | | MYY1791 | Sugar phosphorylase | 55.8 | 2.22E7 | 9.85E6 | 2.26 |  | 3.75E8 | 3.86E8 | 0.97 |
|  | | MYY1406 | Macrolide ABC transporter ATP-binding protein | 25.7 | 7.60E6 | 3.37E6 | 2.25 |  | 1.19E8 | 9.30E7 | 1.28 |
|  | | MYY0516 | Ketol-acid reductoisomerase | 37.3 | 5.63E7 | 2.55E7 | 2.21 |  | 5.45E8 | 3.42E8 | 1.59 |
|  | | MYY1585 | Cell division protein, DivIVA | 30.2 | 3.91E7 | 1.78E7 | 2.20 |  | 9.72E8 | 3.26E8 | 2.98 |
|  | | MYY1447 | Transcription elongation factor, GreA | 17.5 | 1.54E8 | 7.02E7 | 2.19 |  | 1.11E9 | 3.92E8 | 2.84 |
|  | | MYY1427 | Phosphoglucomutase | 62.6 | 9.67E7 | 4.48E7 | 2.16 |  | 1.19E9 | 7.59E8 | 1.57 |
|  | | MYY0795 | S-adenosylmethionine synthetase | 43.1 | 2.22E7 | 1.03E7 | 2.15 |  | 6.93E8 | 4.22E8 | 1.64 |
|  | | MYY1950 | Transketolase | 71.1 | 1.01E8 | 4.75E7 | 2.12 |  | 1.38E9 | 1.41E9 | 0.98 |
|  | | MYY1361 | Purine nucleoside phosphorylase | 26.1 | 4.43E7 | 2.12E7 | 2.10 |  | 4.17E8 | 2.84E8 | 1.47 |
|  | | MYY1261 | Uridylate kinase | 26.4 | 3.00E7 | 1.44E7 | 2.09 |  | 7.47E8 | 4.87E8 | 1.53 |
|  | | MYY0916 | Hypothetical protein MYY0916 | 12.5 | 1.78E7 | 8.56E6 | 2.08 |  | 1.42E8 | 2.51E7 | 5.66 |
|  | | MYY0713 | Glucokinase | 33.4 | 5.78E7 | 2.82E7 | 2.05 |  | 5.80E8 | 3.41E8 | 1.70 |
|  | | MYY1677 | Thioredoxin | 11.4 | 3.99E8 | 1.95E8 | 2.05 |  | 5.11E8 | 3.48E8 | 1.47 |
|  | | MYY1020 | DNA gyrase subunit A | 92.0 | 1.30E7 | 6.36E6 | 2.04 |  | 1.31E8 | 1.35E8 | 0.97 |
|  | | MYY0680 | 50S ribosomal protein L11 | 14.8 | 2.02E8 | 1.00E8 | 2.01 |  | 1.76E9 | 1.54E9 | 1.14 |
| **Significantly decreased in *adr*^S438A^** | | | | | | | | | | | |
|  | MYY1359 | | 30S ribosomal protein S20 | 8.6 | 3.35E8 | 6.73E8 | -2.00 |  | 2.54E9 | 1.93E9 | 1.32 |
|  | MYY0168 | | Membrane protein, PtvC | 40.4 | 5.81E7 | 1.47E8 | -2.25 |  | 7.65E8 | 3.28E8 | 2.33 |

**^a^** The abundance of proteins pulled down by PtvB was detected by LC-MS/MS. Candidate selection standard: |fold change| ≥ 2, score ≥ 5.

**^b^** MW, the molecular weight of the protein in strain ST556.

**^c^** The abundance of each protein was shown as the average of the peak area obtained from 4 repeats in 2 individual experiments.

Gray background indicates the proteins essential for pneumococcal viability (encoded by essential genes).
