# Supplementary material for: Streptococcus pneumoniae synchronizes the states of cell wall peptidoglycan acetylation and genome methylation by programmed DNA inversions
Source: PLoS Pathog. 2025 Aug 5;21(8):e1013286. doi: 10.1371/journal.ppat.1013286 (PMC12324116; doi:10.1371/journal.ppat.1013286)
Supplement: S7 Table — (DOCX) [file ppat.1013286.s013.docx]

**S7 Table. Information of pneumococcal strains used in this study**

| **Strain ID** | **Description** | **Source** |
| --- | --- | --- |
| ST556 | Clinical isolate, serotype 19F; Pen^R^ (4 μg/ml) | From Li et al. [1] |
| ST606 | ST556 derivative; *rpsL1*; Str^R^ | From Li et al. [1] |
| P384 | Clinical isolate, serotype 6A | From Li et al. [1] |
| ST877 | Clinical isolate, serotype 35B | From Li et al. [1] |
| TH6671 | P384 derivative; *rpsL1*; Str^R^ | From Li et al. [1] |
| TH6675 | ST877 derivative; *rpsL1*; Str^R^ | From Li et al. [1] |
| TH6501 | ST606 derivative; ∆*hsdS_A_*::JC; Kan^R^ | From Li et al. [1] |
| TH4533 | D39 derivative; *rpsL1*; Str^R^ | From Wen et al. [2] |
| TH11350 | ST606 derivative; ∆*hk11*::JC1; Kan^R^ | This study |
| TH11861 | ST606 derivative; ∆*hk11*; Str^R^ | This study |
| TH11857 | ST606 derivative; *hk11*^rev-*^ (*pgdA*^-7t→g^); Str^R^ | This study |
| TH13453 | ST606 derivative; *hk11*^rev-*^ ∆*pgdA*^-7t→g^::JC1; Kan^R^ | This study |
| TH13471 | ST606 derivative; *hk11*^rev^; Str^R^ | This study |
| TH6552 | ST606 derivative; *hsdS_A1_* (*psrA*^Y247A^); Str^R^ | From Wang et al. [3] |
| TH13730 | ST606 derivative; ∆*pgdA*::JC1; Kan^R^ | This study |
| TH13734 | ST606 derivative; ∆*pgdA*; Str^R^ | This study |
| TH13742 | ST606 derivative; *pgdA*^D275N^; Str^R^ | This study |
| TH13736 | ST606 derivative; *pgdA*^rev^; Str^R^ | This study |
| TH13732 | ST606 derivative; ∆*adr*::JC1; Kan^R^ | This study |
| TH13738 | ST606 derivative; ∆*adr*; Str^R^ | This study |
| TH14720 | ST606 derivative; *adr*^S438A^; Str^R^ | This study |
| TH13740 | ST606 derivative; *adr*^rev^; Str^R^ | This study |
| TH14279 | TH6671 derivative; ∆*pgdA*::JC1; Kan^R^ | This study |
| TH14281 | TH6671 derivative; *pgdA*^D275N^; Str^R^ | This study |
| TH14283 | TH6671 derivative; *pgdA*^rev^; Str^R^ | This study |
| TH14285 | TH6675 derivative; ∆*pgdA*::JC1; Kan^R^ | This study |
| TH14287 | TH6675 derivative; *pgdA*^D275N^; Str^R^ | This study |
| TH14289 | TH6675 derivative; *pgdA*^rev^; Str^R^ | This study |
| TH14722 | Th6671 derivative; ∆*adr*::JC1; Kan^R^ | This study |
| TH14724 | TH6671 derivative; *adr*^S438A^; Str^R^ | This study |
| TH14726 | TH6671 derivative; *adr*^rev^; Str^R^ | This study |
| TH14728 | TH6675 derivative; ∆*adr*::JC1; Kan^R^ | This study |
| TH14730 | TH6675 derivative; *adr*^S438A^; Str^R^ | This study |
| TH14732 | TH6675 derivative; *adr*^rev^; Str^R^ | This study |

**S7 Table. Information of pneumococcal strains used in this study (Continued)**

| **Strain ID** | **Description** | **Source** |
| --- | --- | --- |
| TH13451 | TH6552 derivative; *hsdS_A1_* ∆*pgdA*::JC1; Kan^R^ | This study |
| TH14570 | TH6552 derivative; *hsdS_A1_ pgdA*^D275N^; Str^R^ | This study |
| TH13467 | TH6552 derivative; *hsdS_A1_ pgdA*^rev^; Str^R^ | This study |
| TH14734 | TH6552 derivative; *hsdS_A1_* ∆*adr*::JC1; Kan^R^ | This study |
| TH14736 | TH6552 derivative; *hsdS_A1_ adr*^S438A^; Str^R^ | This study |
| TH14738 | TH6552 derivative; *hsdS_A1_ adr*^rev^; Str^R^ | This study |
| TH17287 | ST606 derivative; ∆*acoB*::JC1; Kan^R^ | This study |
| TH17288 | ST606 derivative; ∆*acoB*; Str^R^ | This study |
| TH17289 | ST606 derivative; ∆*pfl*::JC1; Kan^R^ | This study |
| TH17290 | ST606 derivative; ∆*pfl*; Str^R^ | This study |
| TH14819 | ST606 derivative; ∆*adr*::JC1 ∆*lytA*; Kan^R^ | This study |
| TH14821 | ST606 derivative; *adr*^S438A^ ∆*lytA*; Str^R^ | This study |
| TH16849 | ST606 derivative; *adr*^S438A^ ∆*lytB*::JC1; Kan^R^ | This study |
| TH16852 | ST606 derivative; *adr*^S438A^ ∆*lytB*; Str^R^ | This study |
| TH16855 | ST606 derivative; *adr*^S438A^ ∆*lytC*::JC1; Kan^R^ | This study |
| TH16858 | ST606 derivative; *adr*^S438A^ ∆*lytC*; Str^R^ | This study |
| TH16861 | ST606 derivative; *adr*^S438A^ ∆*cbpD*::JC1; Kan^R^ | This study |
| TH16864 | ST606 derivative; *adr*^S438A^ ∆*cbpD*; Str^R^ | This study |
| TH7839 | ST606 derivative; ∆*lytA*::JC1; Kan^R^ | This study |
| TH11147 | ST606 derivative; ∆*lytA*; Str^R^ | This study |
| TH16167 | ST606 derivative; *Strep-lytA*; Str^R^ | This study |
| TH14828 | ST606 derivative; *adr*^S438A^ ∆*lytA*::JC1; Kan^R^ | This study |
| TH16192 | ST606 derivative; *adr*^S438A^ *Strep-lytA*; Str^R^ | This study |
| TH16151 | ST606 derivative; *adr*^S438A^ ∆*lytA*^AMI^; Str^R^ | This study |
| TH16846 | ST606 derivative; *adr*^S438A^ ∆*lytA*^CBD^; Str^R^ | This study |
| TH16104 | ST606 derivative; *adr*^S438A^ *lytA*^E87A^; Str^R^ | This study |
| TH16105 | ST606 derivative; *adr*^S438A^ *lytA*^H133A^; Str^R^ | This study |
| TH16152 | ST606 derivative; *adr*^S438A^ *lytA*^S33Q-Y41A^; Str^R^ | This study |
| TH14830 | ST606 derivative; *adr*^S438A^ *lytA*^rev^; Str^R^ | This study |
| TH16871 | ST606 derivative; *adr*^S438A^ ∆*ptvB*::JC1; Kan^R^ | This study |
| TH16873 | ST606 derivative; *adr*^S438A^ ∆*ptvB*; Str^R^ | This study |
| TH17294 | ST606 derivative; *adr*^S438A^ ∆*myy0726*::JC1; Kan^R^ | This study |
| TH17295 | ST606 derivative; *adr*^S438A^ ∆*myy0726*; Str^R^ | This study |

**S7 Table. Information of pneumococcal strains used in this study (Continued)**

| **Strain ID** | **Description** | **Source** |
| --- | --- | --- |
| TH17296 | ST606 derivative; *adr*^S438A^ ∆*myy0914*::JC1; Kan^R^ | This study |
| TH17297 | ST606 derivative; *adr*^S438A^ ∆*myy0914*; Str^R^ | This study |
| TH17298 | ST606 derivative; *adr*^S438A^ ∆*myy1352*::JC1; Kan^R^ | This study |
| TH17299 | ST606 derivative; *adr*^S438A^ ∆*myy1352*; Str^R^ | This study |
| TH17300 | ST606 derivative; *adr*^S438A^ ∆*myy1663*::JC1; Kan^R^ | This study |
| TH17301 | ST606 derivative; *adr*^S438A^ ∆*myy1663*; Str^R^ | This study |
| TH17302 | ST606 derivative; *adr*^S438A^ ∆*myy1707*::JC1; Kan^R^ | This study |
| TH17303 | ST606 derivative; *adr*^S438A^ ∆*myy1707*; Str^R^ | This study |
| TH16378 | ST606 derivative; *adr*^S438A^ ∆*myy2056*(*pcpA*)::JC1; Kan^R^ | This study |
| TH17304 | ST606 derivative; *adr*^S438A^ ∆*myy2056*(*pcpA*) ; Str^R^ | This study |
| TH16874 | ST606 derivative; *adr*^S438A^ *ptvB*^rev^; Str^R^ | This study |
| TH16870 | ST606 derivative; ∆*ptvB*::JC1; Kan^R^ | This study |
| TH16872 | ST606 derivative; ∆*ptvB*; Str^R^ | This study |
| TH17335 | ST606 derivative; *Strep-ptvB*; Str^R^ | This study |
| TH17336 | ST606 derivative; *adr*^S438A^ *Strep-ptvB*; Str^R^ | This study |
| TH17305 | ST606 derivative; *adr*^S438A^ *pcpA*^rev^; Str^R^ | This study |
| TH16371 | ST606 derivative; ∆*pcpA*::JC1; Kan^R^ | This study |
| TH16372 | ST606 derivative; ∆*pcpA*; Str^R^ | This study |
| TH16874 | ST606 derivative; *pcpA*^rev^; Str^R^ | This study |
| TH16866 | ST606 derivative; ∆*ptvA*::JC1; Kan^R^ | This study |
| TH16868 | ST606 derivative; ∆*ptvA*; Str^R^ | This study |
| TH16867 | ST606 derivative; *adr*^S438A^ ∆*ptvA*::JC1; Kan^R^ | This study |
| TH16869 | ST606 derivative; *adr*^S438A^ ∆*ptvA*; Str^R^ | This study |
| TH16875 | ST606 derivative; ∆*ptvC*::JC1; Kan^R^ | This study |
| TH16877 | ST606 derivative; ∆*ptvC*; Str^R^ | This study |
| TH16876 | ST606 derivative; *adr*^S438A^ ∆*ptvC*::JC1; Kan^R^ | This study |
| TH16878 | ST606 derivative; *adr*^S438A^ ∆*ptvC*; Str^R^ | This study |
| TH16879 | ST606 derivative; *adr*^S438A^ *ptvC*^rev^; Str^R^ | This study |
| TH17306 | ST606 derivative; *adr*^S438A^ ∆*myy0041*::*cm^r^*; Cm^R^ | This study |
| TH17307 | ST606 derivative; *adr*^S438A^ ∆*myy0128*::JC1; Kan^R^ | This study |
| TH17308 | ST606 derivative; *adr*^S438A^ ∆*myy0128*; Str^R^ | This study |
| TH17309 | ST606 derivative; *adr*^S438A^ ∆*myy0516*::JC1; Kan^R^ | This study |
| TH17310 | ST606 derivative; *adr*^S438A^ ∆*myy0516*; Str^R^ | This study |
| TH17311 | ST606 derivative; *adr*^S438A^ ∆*hsdM*::JC1; Kan^R^ | This study |

**S7 Table. Information of pneumococcal strains used in this study (Continued)**

| **Strain ID** | **Description** | **Source** |
| --- | --- | --- |
| TH17312 | ST606 derivative; *adr*^S438A^ ∆*hsdM*; Str^R^ | This study |
| TH17313 | ST606 derivative; *adr*^S438A^ ∆*myy0606*::*cm^r^*; Cm^R^ | This study |
| TH17314 | ST606 derivative; *adr*^S438A^ ∆*myy0713*::*cm^r^*; Cm^R^ | This study |
| TH17315 | ST606 derivative; *adr*^S438A^ ∆*myy0734*::*cm^r^*; Cm^R^ | This study |
| TH17316 | ST606 derivative; *adr*^S438A^ ∆*myy0735*::JC1; Kan^R^ | This study |
| TH17317 | ST606 derivative; *adr*^S438A^ ∆*myy0735*; Str^R^ | This study |
| TH17318 | ST606 derivative; *adr*^S438A^ ∆*myy0916*::JC1; Kan^R^ | This study |
| TH17319 | ST606 derivative; *adr*^S438A^ ∆*myy0916*; Str^R^ | This study |
| TH16881 | ST606 derivative; *adr*^S438A^ ∆*dimA*::JC1; Kan^R^ | This study |
| TH16883 | ST606 derivative; *adr*^S438A^ ∆*dimA*; Str^R^ | This study |
| TH17320 | ST606 derivative; *adr*^S438A^ ∆*myy1361*::*cm^r^*; Cm^R^ | This study |
| TH17321 | ST606 derivative; *adr*^S438A^ ∆*myy1406*::*cm^r^*; Cm^R^ | This study |
| TH17322 | ST606 derivative; *adr*^S438A^ ∆*myy1427*::*cm^r^*; Cm^R^ | This study |
| TH17323 | ST606 derivative; *adr*^S438A^ ∆*myy1585*::*cm^r^*; Cm^R^ | This study |
| TH17324 | ST606 derivative; *adr*^S438A^ ∆*myy1791*::JC1; Kan^R^ | This study |
| TH17325 | ST606 derivative; *adr*^S438A^ ∆*myy1791*; Str^R^ | This study |
| TH17326 | ST606 derivative; *adr*^S438A^ ∆*myy1950*::*cm^r^*; Cm^R^ | This study |
| TH16884 | ST606 derivative; *adr*^S438A^ *dimA* ^rev^; Str^R^ | This study |
| TH16880 | ST606 derivative; ∆*dimA*::JC1; Kan^R^ | This study |
| TH16882 | ST606 derivative; ∆*dimA*; Str^R^ | This study |
| TH8422 | ST606 derivative; ∆*ptvR*::JC1; Kan^R^ | This study |
| TH8462 | ST606 derivative; ∆*ptvR*; Str^R^ | This study |
| TH17739 | ST606 derivative; ∆*ptvR* ∆*lytA*::JC1; Kan^R^ | This study |
| TH17740 | ST606 derivative; ∆*ptvR* ∆*lytA*; Str^R^ | This study |
| TH14053 | D39 derivative; ∆*adr*::JC1; Kan^R^ | This study |
| TH14061 | D39 derivative; ∆adr; Str^R^ | This study |
| TH14065 | D39 derivative; *adr*^S438A^; Str^R^ | This study |
| TH17742 | D39 derivative; *adr*^S438A^ ∆*lytA*::JC1; Kan^R^ | This study |
| TH17743 | D39 derivative; *adr*^S438A^ ∆*lytA*; Str^R^ | This study |

**Pen^R^,** penicillin resistance (4 μg/ml); **Cm^R^,** chloramphenicol resistance (4 μg/ml); **Kan^R^,** kanamycin resistance (400 μg/ml); **Str^R^,** streptomycin resistance (150 μg/ml)

**References**

1. Li J, Li JW, Feng Z, Wang J, An H, Liu Y, et al. Epigenetic switch driven by DNA inversions dictates phase variation in *Streptococcus pneumoniae*. PLoS Pathog. 2016; 12(7): e1005762. doi: 10.1371/journal.ppat.1005762. PMID: 27427949.

2. Wen Z, Sertil O, Cheng Y, Zhang S, Liu X, Wang WC, et al. Sequence elements upstream of the core promoter are necessary for full transcription of the capsule gene operon in Streptococcus pneumoniae strain D39. Infect Immun. 2015; 83(5): 1957-1972. doi: 10.1128/IAI.02944-14. PMID: 25733517.

3. Wang J, Li JW, Li J, Huang Y, Wang S, Zhang JR. Regulation of pneumococcal epigenetic and colony phases by multiple two-component regulatory systems. PLoS Pathog. 2020; 16(3): e1008417. doi: 10.1371/journal.ppat.1008417. PMID: 32187228.
