# Supplementary material for: Streptococcus pneumoniae synchronizes the states of cell wall peptidoglycan acetylation and genome methylation by programmed DNA inversions
Source: PLoS Pathog. 2025 Aug 5;21(8):e1013286. doi: 10.1371/journal.ppat.1013286 (PMC12324116; doi:10.1371/journal.ppat.1013286)
Supplement: S8 Table — (DOCX) [file ppat.1013286.s014.docx]

**S8 Table. Information of *E. coli* strains used in this study**

| **Strain ID** | **Description** | **Source** |
| --- | --- | --- |
| DH5α | *recA endA1 gyrA96 thi-1 hsdR17 supE44 relA1* ɸ80 d*lacZ* M15 | Solarbio (Beijing, China) |
| BL21(DE3) | *F–ompT gal dcm lon hsdSB* (*rB-mB-*) *λ* (*DE3* [*lacI lacUV5-T7 gene 1 ind1 sam7 nin5*]) | Solarbio (Beijing, China) |
| TH4805 (BTH101) | Reporter strain for BATCH assay; F^-^ *cya*-99, *ara*D139, *gal*E15, *gal*K16, *rpsL1*, *hsd*R2, *mcr*A1, *mcr*B1 | From Karmova et al. [1] |
| TH7522 | DH5α carrying pKT25; Kan^R^ | This study |
| TH7523 | DH5α carrying pUT18C; Amp^R^ | This study |
| TH7484 | DH5α carrying pUT18; Amp^R^ | This study |
| TH7482 | DH5α carrying pKT25::*zip*; Kan^R^ | From Karmova et al. [1] |
| TH7485 | DH5α carrying pUT18C::*zip*; Amp^R^ | From Karmova et al. [1] |
| TH17327 | DH5α carrying pKT25::*ptvA*; Kan^R^ | This study |
| TH17328 | DH5α carrying pKT25::*ptvB*; Kan^R^ | This study |
| TH17329 | DH5α carrying pUT18C::*ptvB*; Amp^R^ | This study |
| TH17330 | DH5α carrying pUT18C::*ptvC*; Amp^R^ | This study |
| TH17331 | DH5α carrying pUT18C::*ptvBC*; Amp^R^ | This study |
| TH17332 | DH5α carrying pKT25::*ptvC*; Kan^R^ | This study |
| TH17333 | DH5α carrying pUT18C::*dimA*; Amp^R^ | This study |
| TH17334 | DH5α carrying pUT18::*dimA*; Amp^R^ | This study |
| TH16928 | BL21(DE3) carrying pET28a(+)::*lytA*; Kan^R^ | This study |
| TH17732 | DH5α carrying pKT25::*dimA*; Kan^R^ | This study |
| TH17733 | DH5α carrying pKT25::*lytA*; Kan^R^ | This study |
| TH17734 | DH5α carrying pKNT25::*lytA*; Kan^R^ | This study |
| TH17735 | DH5α carrying pUT18::*ptvB*; Amp^R^ | This study |
| TH17736 | DH5α carrying pUT18C::*pcpA*; Amp^R^ | This study |
| TH17737 | DH5α carrying pUT18:: *pcpA*; Amp^R^ | This study |
| TH17738 | DH5α carrying pUT18::*ptvC*; Amp^R^ | This study |
| TH17744 | DH5α carrying pKT25::*psrA*; Kan^R^ | This study |
| TH17745 | DH5α carrying pKNT25::*psrA*; Kan^R^ | This study |

**Kan^R^:** kanamycin resistance (50 μg/ml); **Amp^R^:** ampicillin resistance (100 μg/ml)

**References**

1. Karimova G, Pidoux J, Ullmann A, Ladant D. A bacterial two-hybrid system based on a reconstituted signal transduction pathway. Proc Natl Acad Sci U S A. 1998; 95(10): 5752-5756. doi: 10.1073/pnas.95.10.5752. PMID: 9576956.
