# Supplementary material for: Streptococcus pneumoniae synchronizes the states of cell wall peptidoglycan acetylation and genome methylation by programmed DNA inversions
Source: PLoS Pathog. 2025 Aug 5;21(8):e1013286. doi: 10.1371/journal.ppat.1013286 (PMC12324116; doi:10.1371/journal.ppat.1013286)
Supplement: S9 Table — (DOCX) [file ppat.1013286.s015.docx]

**S9 Table. Information of plasmids used in this study**

| **Plasmids** | **Description** | **Source** |
| --- | --- | --- |
| pIB166 | *E. coli* - *S. pneumoniae* shuttle vector | From Biswas et al. [1] |
| pET28a(+) | *E. coli* protein expression vector | Novagen |
| pTH14588 | pET28a(+)::*lytA* | This study |
| pTH7522 | pKT25 | From Karmova et al. [2] |
| pTH7483 | pKNT25 | From Karmova et al. [2] |
| pTH7523 | pUT18C | From Karmova et al. [2] |
| pTH7484 | pUT18 | From Karmova et al. [2] |
| pTH7482 | pUT18C::*zip* | From Karmova et al. [2] |
| pTH7485 | pKT25::*zip* | From Karmova et al. [2] |
| pTH17333 | pKT25::*ptvA* | This study |
| pTH17334 | pKT25::*ptvB* | This study |
| pTH17335 | pUT18C::*ptvB* | This study |
| pTH17336 | pUT18C::*ptvC* | This study |
| pTH17337 | pUT18C::*ptvBC* | This study |
| pTH17338 | pKT25::*ptvC* | This study |
| pTH17339 | pKT25::*dimA* | This study |
| pTH17340 | pKNT25::*dimA* | This study |
| pTH17733 | pKT25::*lytA* | This study |
| pTH17734 | pKNT25:: *lytA* | This study |
| pTH17735 | pUT18::*ptvB* | This study |
| pTH17736 | pUT18C::*pcpA* | This study |
| pTH17737 | pUT18:: *pcpA* | This study |
| pTH17738 | pUT18::*ptvC* | This study |

**References**

1. Biswas I, Jha JK, Fromm N. Shuttle expression plasmids for genetic studies in *Streptococcus mutans*. Microbiology (Reading). 2008; 154: 2275-2282. doi: 10.1099/mic.0.2008/019265-0. PMID: WOS:000258860200010.

2. Karimova G, Pidoux J, Ullmann A, Ladant D. A bacterial two-hybrid system based on a reconstituted signal transduction pathway. Proc Natl Acad Sci U S A. 1998; 95(10): 5752-5756. doi: 10.1073/pnas.95.10.5752. PMID: 9576956.
