# Supplementary material for: Streptococcus pneumoniae synchronizes the states of cell wall peptidoglycan acetylation and genome methylation by programmed DNA inversions
Source: PLoS Pathog. 2025 Aug 5;21(8):e1013286. doi: 10.1371/journal.ppat.1013286 (PMC12324116; doi:10.1371/journal.ppat.1013286)
Supplement: S10 Table — (DOCX) [file ppat.1013286.s016.docx]

**S10 Table. Primers used for** **mutant construction in this study**

| **Primer ID** | **Sequence (5’-3’)** |
| --- | --- |
| Pr1098 | GAGACTCGAGCCTTTCCTTATGCTTTTGGAC |
| Pr6867 | GAGATCTAGAATTCTACTCCTTATCAATTAAAACAACTC |
| Pr6868 | CAAGGTTTGGCTATGGGCTTG |
| Pr6869 | GAGACTCGAGTAATGGAATGTCTTTCAAATCAGAA |
| Pr6870 | CTTGTATCATAGCCTAATCTTGAAGC |
| Pr8637 | GCTGGATGGCATGGATATTTCTAATAC |
| Pr8638 | ACATCTAGAAATGATAACTCTCCTTCAATTT |
| Pr8639 | ACACTCGAGTTCCTCTCGCCGAAAATCAA |
| Pr8640 | AGCAGACGCTCAAACCATTGTCGCAT |
| Pr8643 | ACATCTAGATTCCTCTCGCCGAAAATCAA |
| Pr9840 | GAGATCTAGAGGATAATGCTGAAAACTCCTTGAAG |
| Pr10985 | GGACTCATCAATTTTTATAAGGAAGATG |
| Pr11098 | ATTTGAAAGACATTCCATTAATTCTACTCCTTATCAATTA |
| Pr11099 | ATTTGAAAGACATTCCATTAATTCTACTCCTTATCAATTA |
| Pr11956 | AGAGGGTATTGACAACAGAAACTTC |
| Pr13543 | ATTATCTATTGTCGGTAGGGATGCC |
| Pr13544 | GCTCTAGACTCGAGCAATCACTACAGGTAGGATGGGAA |
| Pr13545 | CCGCTCGAGAATCAGTCAGAAACATCCAACGGAA |
| Pr13546 | CGATCAGGACAGTCAAATCGATTTC |
| Pr15217 | TAGCATAAATACTCCTTTTCCGATTTT |
| Pr15218 | GCTCTAGA TTCCTCTAGCAAGCCAAAGCATCTTAC |
| Pr15219 | GAGACTCGAG CTCAATACTCGCCTAAAAGCTCATGAG |
| Pr15220 | TAAGCTACGACTTGATCTTGATACACA |
| Pr15221 | CGAGACATGTATTACTCAATACTCGCCTAAAAGCTCATGA |
| Pr15222 | AGGCGAGTATTGAGTAATACATGTCTCGTTTTCCCGTGTC |
| Pr15251 | CATCGACTGCTTTTTTGGCTTCAT |
| Pr15252 | GCTCTAGAATTGCCACAGCTTTGCAGACAGCTCAA |
| Pr15253 | GAGACTCGAGTACCAAAAGTAAACCTATAATCCTAA |

**S10 Table. Primers used for mutant construction in this study (Continued)**

| **Primer ID** | **Sequence (5’-3’)** |
| --- | --- |
| Pr15254 | ACGTTAAGAAATCGATCGAGCGTACC |
| Pr15255 | AAAGCTGTGGCAATTACCAAAAGTAAACCTATAATCCTAA |
| Pr15256 | GTTTACTTTTGGTAATTGCCACAGCTTTGCAGACAGCTCA |
| Pr15480 | TTGCTGGATTTGGACCATCATTAAAGGTTAGAGCGA |
| Pr15481 | AATGATGGTCCAAATCCAGCAA |
| Pr15757 | TGTCACCAATTAACATAGTGCCATC |
| Pr15777 | GATGGCACTATGTTAATTGGTGACACAGTGGCTTTAA |
| Pr18671 | TTCAATCAGTGCAACCGCTGCA |
| Pr18672 | TGCAGCGGTTGCACTGATTGAA |
| Pr18677 | TTCGTGCAATACTCGGCCGTTT |
| Pr18678 | AACGGCCGAGTATTGCACGAA |
| Pr18679 | TTAAACTGCTCACGGCTAATGCCGACTTGAGGCAAATCTGTTCTT |
| Pr18680 | GGCATTAGCCGTGAGCAGTTTAA |
| Pr18681 | ATCCGCTTCATTCTGTACGGTTGAATGC |
| Pr18682 | ACCGTACAGAATGAAGCGGATTATCACTG |
| Pr18683 | GAACTGCGGGTGGCTCCACATATTCTACTCCTTATCAATTAAAAC |
| Pr18684 | TGGAGCCACCCGCAGTTCGAAAAAGAAATTAATGTGAGTAAATTAAGAAC |
| Pr18685 | TCAACCTTGCTTCTCTCCTTGG |
| Pr18686 | GCTCTAGAATCTGCTAGGATTGGTTCATTAGGG |
| Pr18687 | GAGACTCGAGGGTGAGTGGGTAGGATAGGTATCC |
| Pr18688 | CTTCTCCTGTCTGCCGTGTC |
| Pr18689 | GGATACCTATCCTACCCACTCACCATCTGCTAGGATTGGTTCATTAGGG |
| Pr18690 | GGTGAGTGGGTAGGATAGGTATCC |
| Pr19216 | AGCTGGCTTGCTGACTTCTAT |
| Pr19217 | GCTCTAGATTCAGTTCTTGTCATGGCGGAT |
| Pr19218 | GAGACTCGAGCGCTTGGTGCAATTTGTTCTTAAA |
| Pr19219 | TGTCAACGTCTGCTTGGTGTG |

**S10 Table. Primers used for mutant construction in this study (Continued)**

| **Primer ID** | **Sequence (5’-3’)** |
| --- | --- |
| Pr19220 | TTCAGTTCTTGTCATGGCGGAT |
| Pr19221 | ATCCGCCATGACAAGAACTGAACGCTTGGTGCAATTTGTTCTTAAA |
| Pr19223 | AGAGAGTTCCAAGGTCGCAT |
| Pr19224 | GCTCTAGAGATAACGGTAGTCACGACACCAA |
| Pr19225 | GAGACTCGAGACACCAAGCAGACGTTGACAAA |
| Pr19226 | AGCAATGGCAGTGAGGAAGATT |
| Pr19227 | GATAACGGTAGTCACGACACCAA |
| Pr19228 | TTGGTGTCGTGACTACCGTTATCACACCAAGCAGACGTTGACAAA |
| Pr19230 | ACTTAGAGATCACTCTTCACCAGC |
| Pr19231 | GCTCTAGACCATTCTTGTTTCATGGTAGAACCT |
| Pr19232 | GAGACTCGAGGTAGCTGAAGTGGCACTGATTGGA |
| Pr19233 | AGCAATGGCAGTGAGGAAGATT |
| Pr19234 | CCATTCTTGTTTCATGGTAGAACCT |
| Pr19235 | AGGTTCTACCATGAAACAAGAATGGGTAGCTGAAGTGGCACTGATTGGA |
| Pr19237 | TCTTGATTGAAACCTTGGGTAACC |
| Pr19238 | GAGACTCGAGATCGTTCCTCTTTCTAGACTTCTTT |
| Pr19239 | GCTCTAGAGAGTAGAAAGCAAAAATGAACGGTA |
| Pr19240 | CCAGTTCTTTGTCCGTTTGGTC |
| Pr19241 | ATCGTTCCTCTTTCTAGACTTCTTT |
| Pr19242 | AAAGAAGTCTAGAAAGAGGAACGATCCAACAATGAAAGCGAAGTTCG |
| Pr19739 | TTACCTCAAACCACCGTGGAC |
| Pr19740 | GCTCTAGATTTATCTCTTTCTCCTTAGTCTGCGTAAATATCTTCA |
| Pr19741 | GCTCTAGACTCGAGAGTTACATAAGATAAGTTATGTAAATAAAATAAAGCTGACGAGAA |
| Pr19742 | TGATGTATGGGTGTTTCATAAGAGTCTTAACAAC |
| Pr19743 | TCGAGCCTCATAGCTACAGGTAGA |

**S10 Table. Primers used for mutant construction in this study (Continued)**

| **Primer ID** | **Sequence (5’-3’)** |
| --- | --- |
| Pr19744 | GCTCTAGACTCGAGATTAATGTCCTCTTTTTTCTAGTAACAGTCATCTGT |
| Pr19745 | GCTCTAGATCAAGTTCTTGAATAATAAAAAGGGCTCTTTGT |
| Pr19746 | ATTTCAACACTAAGAAAATCATGACCATTCTTGATAAC |
| Pr19747 | GGATTGTAGCAATCGCCTTA |
| Pr19748 | GCTCTAGATGTCATGATTTCCTCTATAGTATTCA |
| Pr19749 | GAGACTCGAGCGTGAGAAAGCTAACAAGAAGAA |
| Pr19750 | GAACGATTAGTCATAATCACAGTAA |
| Pr19751 | TGTCATGATTTCCTCTATAGTATTCA |
| Pr19752 | TGAATACTATAGAGGAAATCATGACACGTGAGAAAGCTAACAAGAAGAA |
| Pr19753 | CCTAGAAGATGCTGGTTTTC |
| Pr19754 | GCTCTAGAATATCTCATACACTCTCCTTATTT |
| Pr19755 | GAGACTCGAGTAAGGAGTTCCTATGGCAAA |
| Pr19756 | CTCGTTGATGTGGATGTTAA |
| Pr19757 | ATATCTCATACACTCTCCTTATTT |
| Pr19758 | AAATAAGGAGAGTGTATGAGATATTAAGGAGTTCCTATGGCAAA |
| Pr19759 | GCTGACTACGCTAACAACTTG |
| Pr19760 | GCTCTAGAGTGCTCTTCCTTTCAGAGTCTT |
| Pr19761 | GAGACTCGAGAACTTGGGAAAGGAGAAGAGT |
| Pr19762 | GCTAATGACGAACCTTGAACA |
| Pr19763 | GTGCTCTTCCTTTCAGAGTCTT |
| Pr19764 | AAGACTCTGAAAGGAAGAGCACAACTTGGGAAAGGAGAAGAGT |
| Pr19765 | ACTTGATTGACCGTTACCAG |
| Pr19766 | GCTCTAGATTCGTTCATTTAATCCTCTTTT |
| Pr19767 | GAGACTCGAGTAGAAGATGAACTCAGTTGTAG |
| Pr19768 | TTGTCCACTTATCCACAATAGC |
| Pr19769 | CTACAACTGAGTTCATCTTCTATTCGTTCATTTAATCCTCTTTT |
| Pr19770 | TAGAAGATGAACTCAGTTGTAG |

**S10 Table. Primers used for mutant construction in this study (Continued)**

| **Primer ID** | **Sequence (5’-3’)** |
| --- | --- |
| Pr19771 | TCGCCAGTAAAGTTAGCAAACTAT |
| Pr19772 | GCTCTAGATTCGTTTGACATATGAAACTCCTT |
| Pr19773 | GAGACTCGAGGAAAACACTCGTGTAAACTAAGAT |
| Pr19774 | CTCTAAATGTCTTCGAGGCAA |
| Pr19775 | TTCGTTTGACATATGAAACTCCTT |
| Pr19776 | AAGGAGTTTCATATGTCAAACGAAGAAAACACTCGTGTAAACTAAGAT |
| Pr19777 | TTTCGAACTGCGGGTGGCTCCAACGTCTGCTTGGTGTGGATA |
| Pr19778 | GAGCCACCCGCAGTTCGAAAAATGACAAAAACGCTTACATCTGCT |
| Pr19779 | ATGAACTTTAATAAAATTGATTTAGACAA |
| Pr19780 | TTATAAAAGCCAGTCATTAGGC |
| Pr19781 | AAGTAGTCGGAGAGATAGAAATC |
| Pr19782 | TTGTCTAAATCAATTTTATTAAAGTTCATTTTTTACCTATCTATTTTTTCTATATAAAGTACA |
| Pr19783 | GCCTAATGACTGGCTTTTATAAAGTAGGTTATGGTTTAGACTACAA |
| Pr19784 | AAGTCAGAACCTGAGATACTAA |
| Pr19785 | TATCCTGACAGCTGATGGAC |
| Pr19786 | GCTCTAGACATTTCTTCTCCTTTTTCCTCT |
| Pr19787 | GAGACTCGAGCTCTCTGTAGAAGATAAGTCCA |
| Pr19788 | TTCATTGACAATAACGTCATCAT |
| Pr19789 | TGGACTTATCTTCTACAGAGAGCATTTCTTCTCCTTTTTCCTCT |
| Pr19790 | CTCTCTGTAGAAGATAAGTCCA |
| Pr19791 | ATGGTGGTTTCCAAATGAC |
| Pr19792 | GCTCTAGACATAGCTTTTCTCTCTTTTCTATTATT |
| Pr19793 | GAGACTCGAGTAATTAGAAATATATAGCGCTGGAG |
| Pr19794 | TCCAATCATATAACCATTCTACCA |
| Pr19795 | CTCCAGCGCTATATATTTCTAATTACATAGCTTTTCTCTCTTTTCTATTATT |
| Pr19796 | TAATTAGAAATATATAGCGCTGGAG |

**S10 Table. Primers used for mutant construction in this study (Continued)**

| **Primer ID** | **Sequence (5’-3’)** |
| --- | --- |
| Pr19797 | GACTCTAATATCCAATCCATGAC |
| Pr19798 | GCTCTAGACAGAATTATCAAGCGGAAAGAG |
| Pr19799 | GAGACTCGAGCATACTTTCCTTTCATTAAGCTACT |
| Pr19800 | AACTTTCAGCATATTCATCTACTT |
| Pr19801 | AGTAGCTTAATGAAAGGAAAGTATGCAGAATTATCAAGCGGAAAGAG |
| Pr19802 | CATACTTTCCTTTCATTAAGCTACT |
| Pr19803 | TTGGGTGATAATGAGCTAACA |
| Pr19804 | TTGTCTAAATCAATTTTATTAAAGTTCATTCTCATCTTCTCTTTCCATACTT |
| Pr19805 | GCCTAATGACTGGCTTTTATAAGAGATAACCTAAAATTAGGCTGT |
| Pr19806 | TACTTCATCAACAACTTCACG |
| Pr19807 | AACTCAAACTGATACAACTTCA |
| Pr19808 | TTGTCTAAATCAATTTTATTAAAGTTCATCATCATATTCTCCTTTTTCATTGT |
| Pr19809 | GCCTAATGACTGGCTTTTATAATTAGCTCTTGCAACTCTAGG |
| Pr19810 | CAATTCCTGAGCTTGTTCAAA |
| Pr19811 | AGCAGATTAAGTCTAACTATTGG |
| Pr19812 | TTGTCTAAATCAATTTTATTAAAGTTCATAGGTTTCTCCTTTTACCTTTAC |
| Pr19813 | GCCTAATGACTGGCTTTTATAAATCAAGGATGAATGGACACA |
| Pr19814 | AAGAAGTGATAAACAAGATAGTTGA |
| Pr19815 | CACTAGTAAAGGTAAAAGGAGAA |
| Pr19816 | GCTCTAGACATGGTATTCTCCTTTTATTTTTCT |
| Pr19817 | GAGACTCGAGGGTTAGGTCAGTAAGATAAAGAG |
| Pr19818 | CTACTAGGCTAATCGGTGAA |
| Pr19819 | CTCTTTATCTTACTGACCTAACCCATGGTATTCTCCTTTTATTTTTCT |
| Pr19820 | GGTTAGGTCAGTAAGATAAAGAG |
| Pr19821 | GTCATTATACTCAGCTTGCCAATCA |
| Pr19822 | GCTCTAGAGTTCTTCTCCTTATTTGATGACGACT |

**S10 Table. Primers used for mutant construction in this study (Continued)**

| **Primer ID** | **Sequence (5’-3’)** |
| --- | --- |
| Pr19823 | GAGACTCGAGGAAAAGAACGATCCTAAAGTCAGGAA |
| Pr19824 | TGGCACGAATGACATCAATAATACC |
| Pr19825 | GTTCTTCTCCTTATTTGATGACGACT |
| Pr19826 | AGTCGTCATCAAATAAGGAGAAGAACGAAAAGAACGATCCTAAAGTCAGGAA |
| Pr19827 | GTAGTCATTCTTGGGAAGGAAA |
| Pr19828 | TTGTCTAAATCAATTTTATTAAAGTTCATCATAATTTATGATACAAAGAGCGAG |
| Pr19829 | GCCTAATGACTGGCTTTTATAAAAGACACAACTGCAGAAGAA |
| Pr19830 | CATCTGAAGAACTTTTCCGTG |
| Pr19831 | GGCAGATCGTCATATCAATGAA |
| Pr19832 | TTGTCTAAATCAATTTTATTAAAGTTCATACTAATTAGTTGCTTCATCAGTC |
| Pr19833 | GCCTAATGACTGGCTTTTATAACCAAACGTCAGATTGTCATTC |
| Pr19834 | TTCTGCAAATACAACGGACT |
| Pr19835 | TGTCTTCAAAGCAGGTAGGA |
| Pr19836 | TTGTCTAAATCAATTTTATTAAAGTTCATTCAAATTCTCCTTTATTTTTAAAACA |
| Pr19837 | GCCTAATGACTGGCTTTTATAAAGACCTATAAAAGATGAGACCA |
| Pr19838 | TGGTTAAGAAGATAAATGAAGCTT |
| Pr19839 | CGGGTCTTGATTTATCTGTT |
| Pr19840 | TTGTCTAAATCAATTTTATTAAAGTTCATCATTCTATTCCTCACTTACTTAAT |
| Pr19841 | GCCTAATGACTGGCTTTTATAACAGATTAAACAGGAAGTGGAAA |
| Pr19842 | TTATCTACTTGTGAAAGAGCGT |
| Pr19843 | GTACCTTTCCAAGTCATCATGA |
| Pr19844 | GCTCTAGACATCATTACTCTCCTTTTTCTAATTGAAG |
| Pr19845 | GAGACTCGAGAATGGAGTTGAAGTAACATTTTGA |
| Pr19846 | TAGATAATTGCTCCAGCGATA |

**S10 Table. Primers used for mutant construction in this study (Continued)**

| **Primer ID** | **Sequence (5’-3’)** |
| --- | --- |
| Pr19847 | TCAAAATGTTACTTCAACTCCATTCATCATTACTCTCCTTTTTCTAATTGAAG |
| Pr19848 | AATGGAGTTGAAGTAACATTTTGA |
| Pr19849 | ATCGATCCATTTGCTATCAAC |
| Pr19850 | TTGTCTAAATCAATTTTATTAAAGTTCATAGAATTTCCTTTCTATTTGAGGTG |
| Pr19851 | GCCTAATGACTGGCTTTTATAATATGGCTTTACTGTCGAAAATC |
| Pr19852 | AACACGTCGATAGGTTTCTT |
| Pr19853 | GGAATTCCATATGATGGAAATTAATGTGAGTAAATTAAGAACA |
| Pr19854 | CCGCTCGAGTTTTACTGTAATCAAGCCATCTGG |
| Pr19855 | ACTCTAGAGGGCGGTGGTGGCTCGGGCGGTGGTGGCTCGGGCGGTGGTGGCTCGATGACAAGAACTGAATACCTGACTCA |
| Pr19856 | TAGGTACCTTACGCATGCTGGTCACC |
| Pr19857 | ACTCTAGAGGGCGGTGGTGGCTCGGGCGGTGGTGGCTCGGGCGGTGGTGGCTCGATGCGTAAATTGACAAAAGGATTTCTCA |
| Pr19858 | TAGGTACCTCAACGTCTGCTTGGTGT |
| Pr19859 | ACTCTAGAGGGCGGTGGTGGCTCGGGCGGTGGTGGCTCGGGCGGTGGTGGCTCGATGAAACAAGAATGGTTTGAAAGT |
| Pr19860 | TCGGTACCTTAAAGAAAGGCCAAGATACGA |
| Pr19861 | GACTCTAGAGGGCGGTGGTGGCTCGGGCGGTGGTGGCTCGGGCGGTGGTGGCTCGATGAACGAAATCAAATGTCCCA |
| Pr19862 | TCGGTACCCTACTCCCCCTTCAGTGCTT |
| Pr19863 | AGAGGATCCCATGAACGAAATCAAATGTCCCA |
| Pr19864 | TCGGTACCCGCGAGCCACCACCGCCCGAGCCACCACCGCCCGAGCCACCACCGCCCTACTCCCCCTTCAGTGCTT |
| P28-F | AGAGGATCGAGATCTCGATCCC |
| P28-R | GCTAGTTATTGCTCAGCGGTGG |
| Pr20146 | ACTCTAGAgggcggtggtggctcgggcggtggtggctcgggcggtggtggctcgatggaaattaatgtgagtaaatt |
| Pr20147 | TAGGTACCttattttactgtaatcaagccat |
| Pr20138 | GCCAAGCTTGatggaaattaatgtgagtaaatt |
| Pr20139 | CTCGGTACCCGcgagccaccaccgcccgagccaccaccgcccgagccaccaccgccttttactgtaatcaagccat |
| Pr20140 | AGAGGATCCCgacgggattaagagcctact |
| Pr20141 | CTCGGTACCCGcgagccaccaccgcccgagccaccaccgcccgagccaccaccgccacgtctgcttggtgtggata |

**S10 Table. Primers used for mutant construction in this study (Continued)**

| **Primer ID** | **Sequence (5’-3’)** |
| --- | --- |
| Pr20144 | AGAGGATCCCggcggtggtggctcgggcggtggtggctcgggcggtggtggctcggatactcctagttcggaagtaatca |
| Pr20145 | CTCGGTACCctatcctacccactcaccg |
| Pr20142 | AGAGGATCCCgatactcctagttcggaagtaatca |
| Pr20143 | CTCGGTACCCGcgagccaccaccgcccgagccaccaccgcccgagccaccaccgcctcctacccactcaccgttag |
|  |  |
