# Supplementary material for: Streptococcus pneumoniae synchronizes the states of cell wall peptidoglycan acetylation and genome methylation by programmed DNA inversions
Source: PLoS Pathog. 2025 Aug 5;21(8):e1013286. doi: 10.1371/journal.ppat.1013286 (PMC12324116; doi:10.1371/journal.ppat.1013286)
Supplement: S11 Table — (DOCX) [file ppat.1013286.s017.docx]

**S11 Table. Construction of bacterial mutants in this study**

| **Strain ID** | **Genotype** | **PCR amplifications for transformation** | | | | **Parental strains** |
| --- | --- | --- | --- | --- | --- | --- |
|  |  | **Target sequence** | **Primer pair** | **Template** | **Digestion/Fusion** |  |
| TH11350 | ST606 ∆*hk11*::JC1 | *hk11*-upstream | Pr13543/ Pr13544 | ST606 | XbaI | ST606 |
|  |  | JC1 | Pr9840/ Pr1098 | TH6501 | XbaI/ XhoI |  |
|  |  | *hk11*-downstream | Pr13545/ Pr13546 | ST606 | XhoI |  |
| TH11861 | ST606 ∆*hk11* | *hk11*-upstream | Pr13543/ Pr13544 | ST606 | XhoI | TH11350 |
|  |  | *hk11*-downstream | Pr13545/ Pr13546 | ST606 | XhoI |  |
| TH11857 | ST606 *hk11*^rev-*^ (*pgdA*^-7t→g^) | *hk11* | Pr13543/ Pr11956 | ST606 | - | TH11350 |
| TH13453 | ST606 *hk11*^rev-*^ ∆*pgdA*^-7t→g^::JC1 | *pgdA*-upstream | Pr15217/15218 | ST606 | XbaI | TH11857 |
|  |  | JC1 | Pr9840/ Pr1098 | TH6501 | XbaI/ XhoI |  |
|  |  | *pgdA*-downstream | Pr15219/ Pr15220 | ST606 | XhoI |  |
| TH13471 | ST606 *hk11*^rev^ | *pgdA* | Pr15217/ Pr15220 | ST606 | - | TH13453 |
| TH13730 | ST606 ∆*pgdA*::JC1 | *pgdA*-upstream | Pr15217/ Pr15218 | ST606 | XbaI | ST606 |
|  |  | JC1 | Pr9840/ Pr1098 | TH6501 | XbaI/ XhoI |  |
|  |  | *pgdA*-downstream | Pr15219/ Pr15220 | ST606 | XhoI |  |
| TH13734 | ST606 ∆*pgdA* | *pgdA*-upstream | Pr15217/ Pr15222 | ST606 | Pr15217/ Pr15220 | TH13730 |
|  |  | *pgdA*-downstream | Pr15221/ Pr15220 | ST606 |  |  |

**S11 Table. Construction of bacterial mutants in this study (Continued)**

| **Strain ID** | **Genotype** | **PCR amplifications for transformation** | | | | **Parental strains** |
| --- | --- | --- | --- | --- | --- | --- |
|  |  | **Target sequence** | **Primer pair** | **Template** | **Digestion/Fusion** |  |
| TH13742 | ST606 *pgdA*^D275N^ | *pgdA*^D275N^-upstream | Pr15217/ Pr15480 | ST606 | Pr15217/ Pr15220 | TH13730 |
|  |  | *pgdA*^D275N^-downstream | Pr15481/ Pr15220 | ST606 |  |  |
| TH13736 | ST606 *pgdA*^rev^ | *pgdA* | Pr15217/ Pr15220 | ST606 | - | TH13730 |
| TH13732 | ST606 ∆*adr*::JC1 | *adr*-upstream | Pr15251/ Pr15252 | ST606 | XbaI | ST606 |
|  |  | JC1 | Pr9840/ Pr1098 | TH6501 | XbaI/ XhoI |  |
|  |  | *adr*-downstream | Pr15253/ Pr15254 | ST606 | XhoI |  |
| TH13738 | ST606 ∆*adr* | *adr*-upstream | Pr15251/ Pr15256 | ST606 | Pr15251/ Pr15254 | TH13732 |
|  |  | *adr*-downstream | Pr15255/ Pr15254 | ST606 |  |  |
| TH14720 | ST606 *adr*^S438A^ | *adr*^S438A^-upstream | Pr15251/ Pr15777 | ST606 | Pr15251/ Pr15254 | TH13732 |
|  |  | *adr*^S438A^-downstream | Pr15757/ Pr15254 | ST606 |  |  |
| TH13740 | ST606 *adr*^rev^ | *adr* | Pr15251/ Pr15254 | ST606 | Pr15251/ Pr15254 | TH13732 |
| TH14279 | TH6671 ∆*pgdA*::JC1 | *pgdA*-upstream | Pr15217/ Pr15218 | TH6671 | XbaI | TH6671 |
|  |  | JC1 | Pr9840/ Pr1098 | TH6501 | XbaI/ XhoI |  |
|  |  | *pgdA*-downstream | Pr15219/ Pr15220 | TH6671 | XhoI |  |
| TH14281 | TH6671 *pgdA*^D275N^ | *pgdA*^D275N^-upstream | Pr15217/ Pr15480 | TH6671 | Pr15217/ Pr15220 | TH14279 |
|  |  | *pgdA*^D275N^-downstream | Pr15481/ Pr15220 | TH6671 |  |  |

**S11 Table. Construction of bacterial mutants in this study (Continued)**

| **Strain ID** | **Genotype** | **PCR amplifications for transformation** | | | | **Parental strains** |
| --- | --- | --- | --- | --- | --- | --- |
|  |  | **Target sequence** | **Primer pair** | **Template** | **Digestion/Fusion** |  |
| TH14283 | TH6671 *pgdA*^rev^ | *pgdA* | Pr15217/ Pr15220 | TH6671 | - | TH14279 |
| TH14285 | TH6675 ∆*pgdA*::JC1 | *pgdA*-upstream | Pr15217/ Pr15218 | TH6675 | XbaI | TH6675 |
|  |  | JC1 | Pr9840/ Pr1098 | TH6501 | XbaI/ XhoI |  |
|  |  | *pgdA*-downstream | Pr15219/ Pr15220 | TH6675 | XhoI |  |
| TH14287 | TH6675 *pgdA*^D275N^ | *pgdA*^D275N^-upstream | Pr15217/ Pr15480 | TH6675 | Pr15217/ Pr15220 | TH14285 |
|  |  | *pgdA*^D275N^-downstream | Pr15481/ Pr15220 | TH6675 |  |  |
| TH14289 | TH6675 *pgdA*^rev^ | *pgdA* | Pr15217/ Pr15220 | TH6675 | - | TH14285 |
| TH14722 | Th6671 ∆*adr*::JC1 | *adr*-upstream | Pr15251/ Pr15252 | TH6671 | XbaI | TH6671 |
|  |  | JC1 | Pr9840/ Pr1098 | TH6501 | XbaI/ XhoI |  |
|  |  | *adr*-downstream | Pr15253/ Pr15254 | TH6671 | XhoI |  |
| TH14724 | TH6671 *adr*^S438A^ | *adr*^S438A^-upstream | Pr15251/ Pr15777 | TH6671 | Pr15251/ Pr15254 | TH14722 |
|  |  | *adr*^S438A^-downstream | Pr15757/ Pr15254 | TH6671 |  |  |
| TH14726 | TH6671 *adr*^rev^ | *adr* | Pr15251/ Pr15254 | TH6671 | Pr15251/ Pr15254 | TH14722 |
| TH14728 | TH6675 ∆*adr*::JC1 | *adr*-upstream | Pr15251/ Pr15252 | TH6675 | XbaI | TH6675 |
|  |  | JC1 | Pr9840/ Pr1098 | TH6501 | XbaI/ XhoI |  |
|  |  | *adr*-downstream | Pr15253/ Pr15254 | TH6675 | XhoI |  |

**S11 Table. Construction of bacterial mutants in this study (Continued)**

| **Strain ID** | **Genotype** | **PCR amplifications for transformation** | | | | **Parental strains** |
| --- | --- | --- | --- | --- | --- | --- |
|  |  | **Target sequence** | **Primer pair** | **Template** | **Digestion/Fusion** |  |
| TH14730 | TH6675 *adr*^S438A^ | *adr*^S438A^-upstream | Pr15251/ Pr15777 | TH6675 | Pr15251/ Pr15254 | TH1728 |
|  |  | *adr*^S438A^-downstream | Pr15757/ Pr15254 | TH6675 |  |  |
| TH14732 | TH6675 *adr*^rev^ | *adr* | Pr15251/ Pr15254 | TH6675 | Pr15251/ Pr15254 | TH14728 |
| TH13451 | TH6552 *hsdS_A1_* ∆*pgdA*::JC1 | *pgdA*-upstream | Pr15217/ Pr15218 | ST606 | XbaI | TH6552 |
|  |  | JC1 | Pr9840/ Pr1098 | TH6501 | XbaI/ XhoI |  |
|  |  | *pgdA*-downstream | Pr15219/ Pr15220 | ST606 | XhoI |  |
| TH14570 | ST606 *hsdS_A1_* *pgdA*^D275N^ | *pgdA*^D275N^-upstream | Pr15217/ Pr15480 | ST606 | Pr15217/ Pr15220 | TH13451 |
|  |  | *pgdA*^D275N^-downstream | Pr15481/ Pr15220 | ST606 |  |  |
| TH13467 | ST606 *hsdS_A1_* *pgdA*^rev^ | *pgdA* | Pr15217/ Pr15220 | ST606 | - | TH13451 |
| TH14734 | ST606 *hsdS_A1_* ∆*adr*::JC1 | *adr*-upstream | Pr15251/ Pr15252 | ST606 | XbaI | TH6552 |
|  |  | JC1 | Pr9840/ Pr1098 | TH6501 | XbaI/ XhoI |  |
|  |  | *adr*-downstream | Pr15253/ Pr15254 | ST606 | XhoI |  |
| TH14736 | ST606 *hsdS_A1_* *adr*^S438A^ | *adr*^S438A^-upstream | Pr15251/ Pr15777 | ST606 | Pr15251/ Pr15254 | TH14734 |
|  |  | *adr*^S438A^-downstream | Pr15757/ Pr15254 | ST606 |  |  |
| TH14738 | ST606 *hsdS_A1_* *adr*^rev^ | *adr* | Pr15251/ Pr15254 | ST606 | Pr15251/ Pr15254 | TH14734 |

**S11 Table. Construction of bacterial mutants in this study (Continued)**

| **Strain ID** | **Genotype** | **PCR amplifications for transformation** | | | | **Parental strains** |
| --- | --- | --- | --- | --- | --- | --- |
|  |  | **Target sequence** | **Primer pair** | **Template** | **Digestion/Fusion** |  |
| TH17287 | ST606 ∆*acoB*::JC1 | *acoB*-upstream | Pr19739/ Pr19740 | ST606 | XbaI | ST606 |
|  |  | JC1 | Pr9840/ Pr1098 | TH6501 | XbaI/ XhoI |  |
|  |  | *acoB*-downstream | Pr19741/ Pr19742 | ST606 | XhoI |  |
| TH17288 | ST606 ∆*acoB* | *acoB*-upstream | Pr19739/ Pr19740 | ST606 | XbaI | TH17287 |
|  |  | *acoB*-downstream | Pr19741/ Pr19742 | ST606 | XbaI |  |
| TH17289 | ST606 ∆*pfl*::JC1 | *pfl*-upstream | Pr19743/ Pr19744 | ST606 | XhoI | ST606 |
|  |  | JC1 | Pr9840/ Pr1098 | TH6501 | XbaI/ XhoI |  |
|  |  | *pfl*-downstream | Pr19745/ Pr19746 | ST606 | XbaI |  |
| TH17290 | ST606 ∆*pfl* | *pfl*-upstream | Pr19743/ Pr19744 | ST606 | XbaI | TH17289 |
|  |  | *pfl*-downstream | Pr19745/ Pr19746 | ST606 | XbaI |  |
| TH14819 | ST606 ∆*adr*::JC1 ∆*lytA* | *adr*-upstream | Pr15251/ Pr15252 | ST606 | XbaI | TH11147 |
|  |  | JC1 | Pr9840/ Pr1098 | TH6501 | XbaI/ XhoI |  |
|  |  | *adr*-downstream | Pr15253/ Pr15254 | ST606 | XhoI |  |
| TH14821 | ST606 *adr*^S438A^ ∆*lytA* | *adr*^S438A^-upstream | Pr15251/ Pr15777 | ST606 | Pr15251/ Pr15254 | TH14819 |
|  |  | *adr*^S438A^-downstream | Pr15757/ Pr15254 | ST606 |  |  |

**S11 Table. Construction of bacterial mutants in this study (Continued)**

| **Strain ID** | **Genotype** | **PCR amplifications for transformation** | | | | **Parental strains** |
| --- | --- | --- | --- | --- | --- | --- |
|  |  | **Target sequence** | **Primer pair** | **Template** | **Digestion/Fusion** |  |
| TH16849 | ST606 *adr*^S438A^ ∆*lytB*::JC1 | *lytB*-upstream | Pr19195/ Pr19196 | ST606 | XbaI | TH14720 |
|  |  | JC1 | Pr9840/ Pr1098 | TH6501 | XbaI/ XhoI |  |
|  |  | *lytB*-downstream | Pr19197/ Pr19198 | ST606 | XhoI |  |
| TH16852 | ST606 *adr*^S438A^ ∆*lytB* | *lytB*-upstream | Pr19195/ Pr19199 | ST606 | Pr19195/ Pr19198 | TH16849 |
|  |  | *lytB*-downstream | Pr19200/ Pr19198 | ST606 |  |  |
| TH16855 | ST606 *adr*^S438A^ ∆*lytC*::JC1 | *lytC*-upstream | Pr19202/ Pr19203 | ST606 | XbaI | TH14720 |
|  |  | JC1 | Pr9840/ Pr1098 | TH6501 | XbaI/ XhoI |  |
|  |  | *lytC*-downstream | Pr19204/ Pr19205 | ST606 | XhoI |  |
| TH16858 | ST606 *adr*^S438A^ ∆*lytC* | *lytC*-upstream | Pr19202/ Pr19206 | ST606 | Pr19202/ Pr19205 | TH16855 |
|  |  | *lytC*-downstream | Pr19207/ Pr19205 | ST606 |  |  |
| TH16861 | ST606 *adr*^S438A^ ∆*cbpD*::JC1 | *cbpD*-upstream | Pr19209/ Pr19210 | ST606 | XbaI | TH14720 |
|  |  | JC1 | Pr9840/ Pr1098 | TH6501 | XbaI/ XhoI |  |
|  |  | *cbpD*-downstream | Pr19211/ Pr19212 | ST606 | XhoI |  |
| TH16864 | ST606 *adr*^S438A^ ∆*cbpD* | *cbpD*-upstream | Pr19209/ Pr19213 | ST606 | Pr19211/ Pr19212 | TH16849 |
|  |  | *cbpD*-downstream | Pr19214/ Pr19212 | ST606 |  |  |

**S11 Table. Construction of bacterial mutants in this study (Continued)**

| **Strain ID** | **Genotype** | **PCR amplifications for transformation** | | | | **Parental strains** |
| --- | --- | --- | --- | --- | --- | --- |
|  |  | **Target sequence** | **Primer pair** | **Template** | **Digestion/Fusion** |  |
| TH7839 | ST606 ∆*lytA*::JC1 | *lytA*-upstream | Pr6868/ Pr6867 | ST606 | XbaI | ST606 |
|  |  | JC1 | Pr9840/ Pr1098 | TH6501 | XbaI/ XhoI |  |
|  |  | *lytA*-downstream | Pr6869/ Pr6870 | ST606 | XhoI |  |
| TH11147 | ST606 ∆*lytA* | *lytA*-upstream | Pr6868/ Pr11098 | ST606 | Pr6868/ Pr6870 | TH7839 |
|  |  | *lytA*-downstream | Pr11099/ Pr6870 | ST606 |  |  |
| TH16167 | ST606 *Strep-lytA* | *Strep-lytA*-upstream | Pr6868/ Pr18683 | ST606 | Pr6868/ Pr6870 | TH7839 |
|  |  | *Strep-lytA*-downstream | Pr18684/ Pr6870 | ST606 |  |  |
| TH14828 | ST606 *adr*^S438A^ ∆*lytA*::JC1 | *lytA*-upstream | Pr6868/ Pr6867 | ST606 | XbaI | TH14720 |
|  |  | JC1 | Pr9840/ Pr1098 | TH6501 | XbaI/ XhoI |  |
|  |  | *lytA*-downstream | Pr6869/ Pr6870 | ST606 | XhoI |  |
| TH16192 | ST606 *adr*^S438A^ *Strep-lytA* | *Strep-lytA*-upstream | Pr6868/ Pr18683 | ST606 | Pr6868/ Pr6870 | TH14828 |
|  |  | *Strep-lytA*-downstream | Pr18684/6870 | ST606 |  |  |
| TH16151 | ST606 *adr*^S438A^ ∆*lytA*^AMI^ | ∆*lytA*^AMI^-upstream | Pr6868/ Pr18679 | ST606 | Pr6868/ Pr6870 | TH14828 |
|  |  | ∆*lytA*^AMI^-downstream | Pr18680/ Pr6870 | ST606 |  |  |
| TH16846 | ST606 *adr*^S438A^ ∆*lytA*^CBD^ | ∆*lytA*^CBD^-upstream | Pr6868/ Pr19189 | ST606 | Pr6868/ Pr6870 | TH14828 |
|  |  | ∆*lytA*^CBD^-downstream | Pr19190/ Pr6870 | ST606 |  |  |

**S11 Table. Construction of bacterial mutants in this study (Continued)**

| **Strain ID** | **Genotype** | **PCR amplifications for transformation** | | | | **Parental strains** |
| --- | --- | --- | --- | --- | --- | --- |
|  |  | **Target sequence** | **Primer pair** | **Template** | **Digestion/Fusion** |  |
| TH16104 | ST606 *adr*^S438A^ *lytA*^E87A^ | *lytA*^E87A^-upstream | Pr6868/ Pr18671 | ST606 | Pr6868/ Pr6870 | TH14828 |
|  |  | *lytA*^E87A^-downstream | Pr18672/ Pr6870 | ST606 |  |  |
| TH16105 | ST606 *adr*^S438A^ *lytA*^H133A^ | *lytA*^H133A^-upstream | Pr6868/ Pr18677 | ST606 | Pr6868/ Pr6870 | TH14828 |
|  |  | *lytA*^H133A^-downstream | Pr18678/ Pr6870 | ST606 |  |  |
| TH16152 | ST606 *adr*^S438A^ *lytA*^S33Q-Y41A^ | *lytA*^S33Q-Y41A^-upstream | Pr6868/ Pr18681 | ST606 | Pr6868/ Pr6870 | TH14828 |
|  |  | *lytA*^S33Q-Y41A^-downstream | Pr18682/ Pr6870 | ST606 |  |  |
| TH14830 | ST606 *adr*^S438A^ *lytA*^rev^ | *lytA* | Pr6868/ Pr6870 | ST606 | - | TH14828 |
| TH16871 | ST606 *adr*^S438A^ ∆*ptvB*::JC1 | *myy0169*-upstream | Pr19223/ Pr19224 | ST606 | XbaI | TH14720 |
|  |  | JC1 | Pr9840/ Pr1098 | TH6501 | XbaI/ XhoI |  |
|  |  | *myy0169*-downstream | Pr19225/ Pr19226 | ST606 | XhoI |  |
| TH16873 | ST606 *adr*^S438A^ ∆*ptvB* | ∆*myy0169*-upstream | Pr19223/ Pr19227 | ST606 | Pr19223/ Pr19226 | TH16871 |
|  |  | ∆*myy0169*-downstream | Pr19228/ Pr19226 | ST606 |  |  |
| TH17294 | ST606 *adr*^S438A^ ∆*myy0726*::JC1 | *myy0726*-upstream | Pr19747/ Pr19748 | ST606 | XbaI | TH14720 |
|  |  | JC1 | Pr9840/ Pr1098 | TH6501 | XbaI/ XhoI |  |
|  |  | *myy0726*-downstream | Pr19749/ Pr19750 | ST606 | XhoI |  |

**S11 Table. Construction of bacterial mutants in this study (Continued)**

| **Strain ID** | **Genotype** | **PCR amplifications for transformation** | | | | **Parental strains** |
| --- | --- | --- | --- | --- | --- | --- |
|  |  | **Target sequence** | **Primer pair** | **Template** | **Digestion/Fusion** |  |
| TH17295 | ST606 *adr*^S438A^ ∆*myy0726* | ∆*myy0726*-upstream | Pr19747/ Pr19751 | ST606 | Pr19747/ Pr19750 | TH17294 |
|  |  | ∆*myy0726*-downstream | Pr19752/ Pr19750 | ST606 |  |  |
| TH17296 | ST606 *adr*^S438A^ ∆*myy0914*::JC1 | *myy0914*-upstream | Pr19753/ Pr19754 | ST606 | XbaI | TH14720 |
|  |  | JC1 | Pr9840/ Pr1098 | TH6501 | XbaI/ XhoI |  |
|  |  | *myy0914*-downstream | Pr19755/ Pr19756 | ST606 | XhoI |  |
| TH17297 | ST606 *adr*^S438A^ ∆*myy0914* | ∆*myy0914*-upstream | Pr19753/ Pr19757 | ST606 | Pr19753/ Pr19756 | TH17296 |
|  |  | ∆*myy0914*-downstream | Pr19758/ Pr19756 | ST606 |  |  |
| TH17298 | ST606 *adr*^S438A^ ∆*myy1352*::JC1 | *myy1352*-upstream | Pr19759/ Pr19760 | ST606 | XbaI | TH14720 |
|  |  | JC1 | Pr9840/ Pr1098 | TH6501 | XbaI/ XhoI |  |
|  |  | *myy1352*-downstream | Pr19761/ Pr19762 | ST606 | XhoI |  |
| TH17299 | ST606 *adr*^S438A^ ∆*myy1352* | ∆*myy1352*-upstream | Pr19759/ Pr19763 | ST606 | Pr19759/ Pr19762 | TH17298 |
|  |  | ∆*myy1352*-downstream | Pr19764/ Pr19762 | ST606 |  |  |
| TH17300 | ST606 *adr*^S438A^ ∆*myy1663*::JC1 | *myy1663*-upstream | Pr19765/ Pr19766 | ST606 | XbaI | TH14720 |
|  |  | JC1 | Pr9840/ Pr1098 | TH6501 | XbaI/ XhoI |  |
|  |  | *myy1663*-downstream | Pr19767/ Pr19768 | ST606 | XhoI |  |

**S11 Table. Construction of bacterial mutants in this study (Continued)**

| **Strain ID** | **Genotype** | **PCR amplifications for transformation** | | | | **Parental strains** |
| --- | --- | --- | --- | --- | --- | --- |
|  |  | **Target sequence** | **Primer pair** | **Template** | **Digestion/Fusion** |  |
| TH17301 | ST606 *adr*^S438A^ ∆*myy1663* | ∆*myy1663*-upstream | Pr19765/ Pr19769 | ST606 | Pr19765/ Pr19768 | TH17300 |
|  |  | ∆*myy1663*-downstream | Pr19770/ Pr19768 | ST606 |  |  |
| TH17302 | ST606 *adr*^S438A^ ∆*myy1707*::JC1 | *myy1707*-upstream | Pr19771/ Pr19772 | ST606 | XbaI | TH14720 |
|  |  | JC1 | Pr9840/ Pr1098 | TH6501 | XbaI/ XhoI |  |
|  |  | *myy1707*-downstream | Pr19773/ Pr19774 | ST606 | XhoI |  |
| TH17303 | ST606 *adr*^S438A^ ∆*myy1707* | ∆*myy1707*-upstream | Pr19771/ Pr19775 | ST606 | Pr19771/ Pr19774 | TH17302 |
|  |  | ∆*myy1707*-downstream | Pr19776/ Pr19774 | ST606 |  |  |
| TH16378 | ST606 *adr*^S438A^ ∆*pcpA*::JC1 | *myy2056*-upstream | Pr18685/ Pr18686 | ST606 | XbaI | TH14720 |
|  |  | JC1 | Pr9840/ Pr1098 | TH6501 | XbaI/ XhoI |  |
|  |  | *myy2056*-downstream | Pr18687/ Pr18688 | ST606 | XhoI |  |
| TH17304 | ST606 *adr*^S438A^ ∆*pcpA* | ∆*myy2056*-upstream | Pr18685/ Pr18689 | ST606 | Pr18685/ Pr18688 | TH16378 |
|  |  | ∆*myy2056*-downstream | Pr18690/ Pr18688 | ST606 |  |  |
| TH16874 | ST606 *adr*^S438A^ *ptvB*^rev^ | *ptvB* | Pr19223/ Pr19226 | ST606 | - | TH16871 |
| TH16870 | ST606 ∆*ptvB*::JC1 | *ptvB*-upstream | Pr19223/ Pr19224 | ST606 | XbaI | ST606 |
|  |  | JC1 | Pr9840/ Pr1098 | TH6501 | XbaI/ XhoI |  |
|  |  | *ptvB*-downstream | Pr19225/ Pr19226 | ST606 | XhoI |  |

**S11 Table. Construction of bacterial mutants in this study (Continued)**

| **Strain ID** | **Genotype** | **PCR amplifications for transformation** | | | | **Parental strains** |
| --- | --- | --- | --- | --- | --- | --- |
|  |  | **Target sequence** | **Primer pair** | **Template** | **Digestion/Fusion** |  |
| TH16872 | ST606 ∆*ptvB* | ∆*ptvB*-upstream | Pr19223/ Pr19227 | ST606 | Pr19223/ Pr19226 | TH16870 |
|  |  | ∆*ptvB*-downstream | Pr19228/ Pr19226 | ST606 |  |  |
| TH17335 | ST606 *Strep-ptvB* | *Strep-ptvB*-upstream | Pr19223/ Pr19777 | ST606 | Pr19223/ Pr19226 | TH16870 |
|  |  | *Strep-ptvB*-downstream | Pr19778/ Pr19226 | ST606 |  |  |
| TH17336 | ST606 *adr*^S438A^ *Strep-ptvB* | *Strep-ptvB*-upstream | Pr19223/ Pr19777 | ST606 | Pr19223/ Pr19226 | TH16871 |
|  |  | *Strep-ptvB*-downstream | Pr19778/ Pr19226 | ST606 |  |  |
| TH17305 | ST606 *adr*^S438A^ *pcpA*^rev^ | *pcpA* | Pr18685/ Pr18688 | ST606 | - | TH16378 |
| TH16371 | ST606 ∆*pcpA*::JC1 | *pcpA*-upstream | Pr18685/ Pr18686 | ST606 | XbaI | ST606 |
|  |  | JC1 | Pr9840/ Pr1098 | TH6501 | XbaI/ XhoI |  |
|  |  | *pcpA*-downstream | Pr18687/ Pr18688 | ST606 | XhoI |  |
| TH16372 | ST606 ∆*pcpA* | ∆*pcpA*-upstream | Pr18685/ Pr18689 | ST606 | Pr18685/ Pr18688 | TH16371 |
|  |  | ∆*pcpA*-downstream | Pr18690/ Pr18688 | ST606 |  |  |
| TH16874 | ST606 *pcpA*^rev^ | *pcpA* | Pr18685/ Pr18688 | ST606 | - | TH16371 |
| TH16866 | ST606 ∆*ptvA*::JC1 | *ptvA*-upstream | Pr19216/19217 | ST606 | XbaI | ST606 |
|  |  | JC1 | Pr9840/ Pr1098 | TH6501 | XbaI/ XhoI |  |
|  |  | *ptvA*-downstream | Pr19218/19219 | ST606 | XhoI |  |

**S11 Table. Construction of bacterial mutants in this study (Continued)**

| **Strain ID** | **Genotype** | **PCR amplifications for transformation** | | | | **Parental strains** |
| --- | --- | --- | --- | --- | --- | --- |
|  |  | **Target sequence** | **Primer pair** | **Template** | **Digestion/Fusion** |  |
| TH16868 | ST606 ∆*ptvA* | ∆*ptvA*-upstream | Pr19216/ Pr19220 | ST606 | Pr19216/ Pr19219 | TH16866 |
|  |  | ∆*ptvA*-downstream | Pr19221/ Pr19219 | ST606 |  |  |
| TH16867 | ST606 *adr*^S438A^ ∆*ptvA*::JC1 | *ptvA*-upstream | Pr19216/ Pr19217 | ST606 | XbaI | TH14720 |
|  |  | JC1 | Pr9840/ Pr1098 | TH6501 | XbaI/ XhoI |  |
|  |  | *ptvA*-downstream | Pr19218/ Pr19219 | ST606 | XhoI |  |
| TH16869 | ST606 *adr*^S438A^ ∆*ptvA* | ∆*ptvA*-upstream | Pr19216/ Pr19220 | ST606 | Pr19216/ Pr19219 | TH16867 |
|  |  | ∆*ptvA*-downstream | Pr19221/ Pr19219 | ST606 |  |  |
| TH16875 | ST606 ∆*ptvC*::JC1 | *ptvC*-upstream | Pr19230/ Pr19231 | ST606 | XbaI | ST606 |
|  |  | JC1 | Pr9840/ Pr1098 | TH6501 | XbaI/ XhoI |  |
|  |  | *ptvC*-downstream | Pr19232/ Pr19233 | ST606 | XhoI |  |
| TH16877 | ST606 ∆*ptvC* | ∆*ptvC*-upstream | Pr19230/ Pr19234 | ST606 | Pr19230/ Pr19233 | TH16875 |
|  |  | ∆*ptvC*-downstream | Pr19235/ Pr19233 | ST606 |  |  |
| TH16876 | ST606 *adr*^S438A^ ∆*ptvC*::JC1 | *ptvC*-upstream | Pr19230/ Pr19231 | ST606 | XbaI | TH14720 |
|  |  | JC1 | Pr9840/ Pr1098 | TH6501 | XbaI/ XhoI |  |
|  |  | *ptvC*-downstream | Pr19232/ Pr19233 | ST606 | XhoI |  |

**S11 Table. Construction of bacterial mutants in this study (Continued)**

| **Strain ID** | **Genotype** | **PCR amplifications for transformation** | | | | **Parental strains** |
| --- | --- | --- | --- | --- | --- | --- |
|  |  | **Target sequence** | **Primer pair** | **Template** | **Digestion/Fusion** |  |
| TH16878 | ST606 *adr*^S438A^ ∆*ptvC* | ∆*ptvC*-upstream | Pr19230/ Pr19234 | ST606 | Pr19230/ Pr19233 | TH16875 |
|  |  | ∆*ptvC*-downstream | Pr19235/ Pr19233 | ST606 |  |  |
| TH16879 | ST606 *adr*^S438A^ *ptvC*^rev^ | *ptvC* | Pr19230/ Pr19233 | ST606 | - | TH16875 |
| TH17306 | ST606 *adr*^S438A^ ∆*myy0041*::*cm^r^* | *myy0041*-upstream | Pr19781/ Pr19782 | ST606 | Pr19781/ Pr19784 | TH14720 |
|  |  | *cm^r^* | Pr19779/ Pr19780 | pIB166 |  |  |
|  |  | *myy0041*-downstream | Pr19783/ Pr19784 | ST606 |  |  |
| TH17307 | ST606 *adr*^S438A^ ∆*myy0128*::JC1 | *myy0128*-upstream | Pr19785/ Pr19786 | ST606 | XbaI | TH14720 |
|  |  | JC1 | Pr9840/ Pr1098 | TH6501 | XbaI/ XhoI |  |
|  |  | *myy0128*-downstream | Pr19787/ Pr19788 | ST606 | XhoI |  |
| TH17308 | ST606 *adr*^S438A^ ∆*myy0128* | ∆*myy0128*-upstream | Pr19785/ Pr19789 | ST606 | Pr19785/ Pr19788 | TH17307 |
|  |  | ∆*myy0128*-downstream | Pr19790/ Pr19788 | ST606 |  |  |
| TH17309 | ST606 *adr*^S438A^ ∆*myy0516*::JC1 | *myy0516*-upstream | Pr19791/ Pr19792 | ST606 | XbaI | TH14720 |
|  |  | JC1 | Pr9840/ Pr1098 | TH6501 | XbaI/ XhoI |  |
|  |  | *myy0516*-downstream | Pr19793/ Pr19794 | ST606 | XhoI |  |

**S11 Table. Construction of bacterial mutants in this study (Continued)**

| **Strain ID** | **Genotype** | **PCR amplifications for transformation** | | | | **Parental strains** |
| --- | --- | --- | --- | --- | --- | --- |
|  |  | **Target sequence** | **Primer pair** | **Template** | **Digestion/Fusion** |  |
| TH17310 | ST606 *adr*^S438A^ ∆*myy0516* | ∆*myy0516*-upstream | Pr19791/ Pr19795 | ST606 | Pr19791/ Pr19794 | TH17309 |
|  |  | ∆*myy0516*-downstream | Pr19796/ Pr19794 | ST606 |  |  |
| TH17311 | ST606 *adr*^S438A^ ∆*hsdM*::JC1 | *hsdM*-upstream | Pr19797/ Pr19798 | ST606 | XbaI | TH14720 |
|  |  | JC1 | Pr9840/ Pr1098 | TH6501 | XbaI/ XhoI |  |
|  |  | *hsdM*-downstream | Pr19799/ Pr19800 | ST606 | XhoI |  |
| TH17312 | ST606 *adr*^S438A^ ∆*hsdM* | ∆*hsdM*-upstream | Pr19797/ Pr19801 | ST606 | Pr19797/ Pr19800 | TH17311 |
|  |  | ∆*hsdM*-downstream | Pr19802/ Pr19800 | ST606 |  |  |
| TH17313 | ST606 *adr*^S438A^ ∆*myy0606*::*cm^r^* | *myy0606*-upstream | Pr19803/ Pr19804 | ST606 | Pr19803/ Pr19806 | TH14720 |
|  |  | *cm^r^* | Pr19779/ Pr19780 | pIB166 |  |  |
|  |  | *myy0606*-downstream | Pr19805/ Pr19806 | ST606 |  |  |
| TH17314 | ST606 *adr*^S438A^ ∆*myy0713*::*cm^r^* | *myy0713*-upstream | Pr19807/ Pr19808 | ST606 | Pr19807/ Pr19810 | TH14720 |
|  |  | *cm^r^* | Pr19779/ Pr19780 | pIB166 |  |  |
|  |  | *myy0713*-downstream | Pr19809/ Pr19810 | ST606 |  |  |
| TH17315 | ST606 *adr*^S438A^ ∆*myy0734*::*cm^r^* | *myy0734*-upstream | Pr19811/ Pr19812 | ST606 | Pr19811/ Pr19814 | TH14720 |
|  |  | *cm^r^* | Pr19779/ Pr19780 | pIB166 |  |  |
|  |  | *myy0734*-downstream | Pr19813/ Pr19814 | ST606 |  |  |

**S11 Table. Construction of bacterial mutants in this study (Continued)**

| **Strain ID** | **Genotype** | **PCR amplifications for transformation** | | | | **Parental strains** |
| --- | --- | --- | --- | --- | --- | --- |
|  |  | **Target sequence** | **Primer pair** | **Template** | **Digestion/Fusion** |  |
| TH17316 | ST606 *adr*^S438A^ ∆*myy0735*::JC1 | *myy0735*-upstream | Pr19815/ Pr19816 | ST606 | XbaI | TH14720 |
|  |  | JC1 | Pr9840/ Pr1098 | TH6501 | XbaI/ XhoI |  |
|  |  | *myy0735*-downstream | Pr19817/ Pr19818 | ST606 | XhoI |  |
| TH17317 | ST606 *adr*^S438A^ ∆*myy0735* | ∆*myy0735*-upstream | Pr19815/ Pr19819 | ST606 | Pr19815/ Pr19818 | TH17316 |
|  |  | ∆*myy0735*-downstream | Pr19820/ Pr19818 | ST606 |  |  |
| TH17318 | ST606 *adr*^S438A^ ∆*myy0916*::JC1 | *myy0916*-upstream | Pr19821/ Pr19822 | ST606 | XbaI | TH14720 |
|  |  | JC1 | Pr9840/ Pr1098 | TH6501 | XbaI/ XhoI |  |
|  |  | *myy0916*-downstream | Pr19823/ Pr19824 | ST606 | XhoI |  |
| TH17319 | ST606 *adr*^S438A^ ∆*myy0916* | ∆*myy0916*-upstream | Pr19821/ Pr19825 | ST606 | Pr19821/ Pr19824 | TH17318 |
|  |  | ∆*myy0916*-downstream | Pr19826/ Pr19824 | ST606 |  |  |
| TH16881 | ST606 *adr*^S438A^ ∆*dimA*::JC1 | *dimA*-upstream | Pr19237/ Pr19238 | ST606 | XbaI | TH14720 |
|  |  | JC1 | Pr9840/ Pr1098 | TH6501 | XbaI/ XhoI |  |
|  |  | *dimA*-downstream | Pr19239/ Pr19240 | ST606 | XhoI |  |
| TH16883 | ST606 *adr*^S438A^ ∆*dimA* | ∆ *dimA*-upstream | Pr19237/ Pr19241 | ST606 | Pr19237/ Pr19240 | TH16881 |
|  |  | ∆ *dimA*-downstream | Pr19242/ Pr19240 | ST606 |  |  |

**S11 Table. Construction of bacterial mutants in this study (Continued)**

| **Strain ID** | **Genotype** | **PCR amplifications for transformation** | | | | **Parental strains** |
| --- | --- | --- | --- | --- | --- | --- |
|  |  | **Target sequence** | **Primer pair** | **Template** | **Digestion/Fusion** |  |
| TH16883 | ST606 *adr*^S438A^ ∆*dimA* | ∆*myy1025*-upstream | Pr19237/ Pr19241 | ST606 | Pr19237/ Pr19240 | TH16881 |
|  |  | ∆*myy1025*-downstream | Pr19242/ Pr19240 | ST606 |  |  |
| TH17320 | ST606 *adr*^S438A^ ∆*myy1361*::*cm^r^* | *myy1361*-upstream | Pr19827/ Pr19828 | ST606 | Pr19827/ Pr19830 | TH14720 |
|  |  | *cm^r^* | Pr19779/ Pr19780 | pIB166 |  |  |
|  |  | *myy1361*-downstream | Pr19829/ Pr19830 | ST606 |  |  |
| TH17321 | ST606 *adr*^S438A^ ∆*myy1406*::*cm^r^* | *myy1406*-upstream | Pr19831/ Pr19832 | ST606 | Pr19831/ Pr19834 | TH14720 |
|  |  | *cm^r^* | Pr19779/ Pr19780 | pIB166 |  |  |
|  |  | *myy1406*-downstream | Pr19833/ Pr19834 | ST606 |  |  |
| TH17322 | ST606 *adr*^S438A^ ∆*myy1427*::*cm^r^* | *myy1427*-upstream | Pr19835/ Pr19836 | ST606 | Pr19835/ Pr19838 | TH14720 |
|  |  | *cm^r^* | Pr19779/ Pr19780 | pIB166 |  |  |
|  |  | *myy1427*-downstream | Pr19837/ Pr19838 | ST606 |  |  |
| TH17323 | ST606 *adr*^S438A^ ∆*myy1585*::*cm^r^* | *myy1585*-upstream | Pr19839/ Pr19840 | ST606 | Pr19839/ Pr19842 | TH14720 |
|  |  | *cm^r^* | Pr19779/ Pr19780 | pIB166 |  |  |
|  |  | *myy1585*-downstream | Pr19841/ Pr19842 | ST606 |  |  |

**S11 Table. Construction of bacterial mutants in this study (Continued)**

| **Strain ID** | **Genotype** | **PCR amplifications for transformation** | | | | **Parental strains** |
| --- | --- | --- | --- | --- | --- | --- |
|  |  | **Target sequence** | **Primer pair** | **Template** | **Digestion/Fusion** |  |
| TH17324 | ST606 *adr*^S438A^ ∆*myy1791*::JC1 | *myy1791*-upstream | Pr19843/ Pr19844 | ST606 | XbaI | TH14720 |
|  |  | JC1 | Pr9840/ Pr1098 | TH6501 | XbaI/ XhoI |  |
|  |  | *myy1791*-downstream | Pr19845/ Pr19846 | ST606 | XhoI |  |
| TH17325 | ST606 *adr*^S438A^ ∆*myy1791* | ∆*myy1791*-upstream | Pr19843/ Pr19847 | ST606 | Pr19843/ Pr19846 | TH17324 |
|  |  | ∆*myy1791*-downstream | Pr19848/ Pr19846 | ST606 |  |  |
| TH17326 | ST606 *adr*^S438A^ ∆*myy1950*::*cm^r^* | *myy1950*-upstream | Pr19849/ Pr19850 | ST606 | Pr19849/ Pr19852 | TH14720 |
|  |  | *cm^r^* | Pr19779/ Pr19780 | pIB166 |  |  |
|  |  | *dimA*-downstream | Pr19851/ Pr19852 | ST606 |  |  |
| TH16880 | ST606 ∆*dimA*::JC1 | *dimA*-upstream | Pr19237/ Pr19238 | ST606 | XbaI | ST606 |
|  |  | JC1 | Pr9840/ Pr1098 | TH6501 | XbaI/ XhoI |  |
|  |  | *dimA*-downstream | Pr19239/ Pr19240 | ST606 | XhoI |  |
| TH16882 | ST606 ∆*dimA* | ∆*dimA*-upstream | Pr19237/ Pr19241 | ST606 | Pr19237/ Pr19240 | TH16880 |
|  |  | ∆*dimA*-downstream | Pr19242/ Pr19240 | ST606 |  |  |
| TH16884 | ST606 *adr*^S438A^ *dimA*^rev^ | *dimA* | Pr19237/ Pr19240 | ST606 | - | TH16881 |
| TH8422 | ST606 ∆*ptvR*::JC1 | *myy0171-*upstream | Pr11501/ Pr11502 | ST606 | XbaI | ST606 |
|  |  | JC1 | Pr9840/ Pr1098 | TH6501 | XbaI/ XhoI |  |
|  |  | *myy0171-*downstream | Pr11503/ Pr11504 | ST606 | XhoI |  |
| TH8462 | ST606 ∆*ptvR* | ∆*myy0171-*downstream | Pr11505/ Pr11504 | ST606 | Pr11501/ Pr11504 | TH8422 |
|  |  | ∆*myy0171-*upstream | Pr11501/ Pr11506 | ST606 |  |  |

**S11 Table. Construction of bacterial mutants in this study (Continued)**

| **Strain ID** | **Genotype** | **PCR amplifications for transformation** | | | | **Parental strains** |
| --- | --- | --- | --- | --- | --- | --- |
|  |  | **Target sequence** | **Primer pair** | **Template** | **Digestion/Fusion** |  |
| TH17739 | ST606 ∆*ptvR* ∆*lytA*::JC1 | *lytA*-upstream | Pr6868/ Pr6867 | ST606 | XbaI | TH8422 |
|  |  | JC1 | Pr9840/ Pr1098 | TH6501 | XbaI/ XhoI |  |
|  |  | *lytA*-downstream | Pr6869/ Pr6870 | ST606 | XhoI |  |
| TH17740 | ST606 ∆*ptvR* ∆*lytA* | *lytA*-upstream | Pr6868/ Pr11098 | ST606 | Pr6868/ Pr6870 | TH17739 |
|  |  | *lytA*-downstream | Pr11099/ Pr6870 | ST606 |  |  |
| TH14053 | D39 ∆*adr*::JC1 | *adr*-upstream | Pr15251/ Pr15252 | TH4533 | XbaI | TH4533 |
|  |  | JC1 | Pr9840/ Pr1098 | TH6501 | XbaI/ XhoI |  |
|  |  | *adr*-downstream | Pr15253/ Pr15254 | TH4533 | XhoI |  |
| TH14061 | D39 ∆*adr* | *adr*-upstream | Pr15251/ Pr15256 | TH4533 | XbaI | TH14053 |
|  |  | *adr*-downstream | Pr15595/ Pr1525 | TH4533 | XhoI |  |
| TH14065 | D39 *adr*^S438A^ | *adr*^S438A^-upstream | Pr15251/ Pr15777 | TH4533 | Pr15251/ Pr15254 | TH14053 |
|  |  | *adr*^S438A^-downstream | Pr15757/ Pr15254 | TH4533 |  |  |
| TH17742 | D39 *adr*^S438A^ ∆*lytA*::JC1 | *lytA*-upstream | Pr6868/ Pr6867 | ST606 | XbaI | TH14065 |
|  |  | JC1 | Pr9840/ Pr1098 | TH6501 | XbaI/ XhoI |  |
|  |  | *lytA*-downstream | Pr6869/ Pr6870 | ST606 | XhoI |  |
| TH17743 | D39 *adr*^S438A^ ∆*lytA* | *lytA*-upstream | Pr6868/ Pr11098 | ST606 | Pr6868/ Pr6870 | TH17742 |
|  |  | *lytA*-downstream | Pr11099/ Pr6870 | ST606 |  |  |
| TH17327 | DH5α pKT25::*ptvA* | pKT25 | - | pKT25 | XbaI/ KpnI | DH5α |
|  |  | *ptvA* | Pr19855/ Pr19856 | ST606 | XbaI/ KpnI |  |

**S11 Table. Construction of bacterial mutants in this study (Continued)**

| **Strain ID** | **Genotype** | **PCR amplifications for transformation** | | | | **Parental strains** |
| --- | --- | --- | --- | --- | --- | --- |
|  |  | **Target sequence** | **Primer pair** | **Template** | **Digestion/Fusion** |  |
| TH17328 | DH5α pKT25::*ptvB* | pKT25 | - | pKT25 | XbaI/ KpnI | DH5α |
|  |  | *ptvB* | Pr19857/ Pr19858 | ST606 | XbaI/ KpnI |  |
| TH17329 | DH5α pUT18C::*ptvB* | pUT18C | - | pUT18C | XbaI/ KpnI | DH5α |
|  |  | *ptvB* | Pr19857/ Pr19858 | ST606 | XbaI/ KpnI |  |
| TH17330 | DH5α pUT18C::*ptvC* | pUT18C | - | pUT18C | XbaI/ KpnI | DH5α |
|  |  | *ptvC* | Pr19859/ Pr19860 | ST606 | XbaI/ KpnI |  |
| TH17331 | DH5α pUT18C::*ptvBC* | pUT18C | - | pUT18C | XbaI/ KpnI | DH5α |
|  |  | *ptvB-ptvC* | Pr19857/ Pr19860 | ST606 | XbaI/ KpnI |  |
| TH17332 | DH5α pKT::*ptvC* | pKT25 | - | pKT25 | XbaI/ KpnI | DH5α |
|  |  | *ptvC* | Pr19859/ Pr19860 | ST606 | XbaI/ KpnI |  |
| TH17333 | DH5α pUT18C::*dimA* | pUT18C | - | pUT18C | XbaI/ KpnI | DH5α |
|  |  | *dimA* | Pr19861/ Pr19862 | ST606 | XbaI/ KpnI |  |
| TH17334 | DH5α pUT18::*dimA* | pUT18 | - | pUT18 | BamHI/ KpnI | DH5α |
|  |  | *dimA* | Pr19863/ Pr19864 | ST606 | BamHI/ KpnI |  |
| TH16928 | BL21(DE3) pET28a(+)::*lytA* | pET28a(+) | - | pET28a(+) | XhoI/NdeI | BL21(DE3) |
|  |  | *lytA* | Pr19853/ Pr19854 | ST606 | XhoI/NdeI |  |
| TH17732 | DH5α pKT25:: *dimA* | pKT25 | - | pKT25 | XbaI/ KpnI | DH5α |
|  |  | *dimA* | Pr19861/ Pr19862 | ST606 | XbaI/ KpnI |  |
| TH17733 | DH5α pKT25::*lytA* | pKT25 | - | pKT25 | XbaI/ KpnI | DH5α |
|  |  | *lytA* | Pr20146/ Pr20147 | ST606 | XbaI/ KpnI |  |
| TH17734 | DH5α pKNT25::*lytA* | pKNT25 | - | pKNT25 | HindIII/ KpnI | DH5α |
|  |  | *lytA* | Pr20138/ Pr20139 | ST606 | HindIII/ KpnI |  |

**S11 Table. Construction of bacterial mutants in this study (Continued)**

| **Strain ID** | **Genotype** | **PCR amplifications for transformation** | | | | **Parental strains** |
| --- | --- | --- | --- | --- | --- | --- |
|  |  | **Target sequence** | **Primer pair** | **Template** | **Digestion/Fusion** |  |
| TH17735 | DH5α pUT18::*ptvB* | pUT18 | - | pUT18 | BamHI/ KpnI | DH5α |
|  |  | *ptvB* | Pr20140/ Pr20141 | ST606 | BamHI/ KpnI |  |
| TH17736 | DH5α pUT18C::*pcpA* | pUT18C | - | pUT18C | XbaI/ KpnI | DH5α |
|  |  | *pcpA* | Pr20144/ Pr20145 | ST606 | XbaI/ KpnI |  |
| TH17737 | DH5α pUT18:: *pcpA* | pUT18 | - | pUT18 | BamHI/ KpnI | DH5α |
|  |  | *pcpA* | Pr20142/ Pr20143 | ST606 | BamHI/ KpnI |  |
| TH17738 | DH5α pUT18::*ptvC* | pUT18 | - | pUT18 | BamHI/ KpnI | DH5α |
|  |  | *ptvC* | Pr19859/ Pr19860 | ST606 | BamHI/ KpnI |  |
| TH17744 | DH5α pKT25::*psrA* | pKT25 | - | pKT25 | XbaI/ KpnI | DH5α |
|  |  | *psrA* | Pr20148/ Pr20149 | ST606 | XbaI/ KpnI |  |
| TH17745 | DH5α pKNT25::*psrA* | pKNT25 | - | pKNT25 | HindIII/ KpnI | DH5α |
|  |  | *psrA* | Pr20150/ Pr20151 | ST606 | HindIII/ KpnI | DH5α |
