# Supplementary material for: Streptococcus pneumoniae synchronizes the states of cell wall peptidoglycan acetylation and genome methylation by programmed DNA inversions
Source: PLoS Pathog. 2025 Aug 5;21(8):e1013286. doi: 10.1371/journal.ppat.1013286 (PMC12324116; doi:10.1371/journal.ppat.1013286)
Supplement: S12 Table — (DOCX) [file ppat.1013286.s018.docx]

**S12 Table.** **Primers used for qRT-PCR and qPCR in this study**

| **Primer ID** | **Sequence (5’-3’)** | **Application** |
| --- | --- | --- |
| P1 | TGAGTGGCAGGAATATCCAATATGGC | Detection of IR1-mediated inversion |
| P2 | ATCCACCAGCCACAACACCATCATAG |  |
| P3 | CGAATTTATCTAAGGAAAACAGCTACTGAACAAC |  |
|  |  |  |
| P4 | CTACTGGAACAAGTTATCCTGCAATCAATG | Detection of IR2-mediated inversion |
| P5 | AAAGTTGCTTCTATTCTTATCCCTCTCCCTC |  |
| P6 | CCAACTTTCTGGTATTTCACAAGGTACTTCC |  |
|  |  |  |
| P7 | CATCCATCGTGTATTGCACCATCAAC | Detection of IR3-mediated inversion |
| P8 | TCGTTAAGTAAAGTATCCAAAACAATAAATGCTG |  |
| P9 | AAAACTTATCAGTGAAGGAAAAATCAAACGAG |  |
|  |  |  |
| Pr7932 | GATTGCCATCATGAGTGACAAGG | Detection of *era* abundance |
| Pr7933 | AGTGTCCACTTCGCGAAGGGT |  |
|  |  |  |
| Pr16178 | GACACCAGAACAACTTAAAGCAAGT | Detection of *hsdS* mRNA abundance |
| Pr16179 | CACTCCCAAGTATCAGGAATATCAT |  |
|  |  |  |
| Pr16174 | GTTGCTTCTATTCTTATCCCTCTCC | Detection of *hsdS_A1_* mRNA abundance |
| Pr16175 | TTTTTATAACAACCCAATTCATAGGT |  |
|  |  |  |
| Pr19737 | CGTGTCCCATCAAATCTGCAAG | Detection of *psrA* mRNA abundance |
| Pr19738 | ACGAACCAATTACAGCAAGAGGA |  |
|  |  |  |
| Pr16174 | GTTGCTTCTATTCTTATCCCTCTCC | Detection of *hsdS_A2_* mRNA abundance |
| Pr20158 | ccatgcttctgggatttcat |  |

**S12 Table. Primers used for qRT-PCR and qPCR in this study**

| **Primer ID** | **Sequence (5’-3’)** | **Application** |
| --- | --- | --- |
| Pr16174 | GTTGCTTCTATTCTTATCCCTCTCC | Detection of *hsdS_A3_* mRNA abundance |
| Pr20159 | cacccattcccaactttctg |  |
|  |  |  |
| Pr20159 | cacccattcccaactttctg | Detection of *hsdS_A4_* mRNA abundance |
| Pr20160 | ccccactatccgaacaacaa |  |
|  |  |  |
| Pr20158 | ccatgcttctgggatttcat | Detection of *hsdS_A5_* mRNA abundance |
| Pr20160 | ccccactatccgaacaacaa |  |
|  |  |  |
| Pr16175 | TTTTTATAACAACCCAATTCATAGGT | Detection of *hsdS_A6_* mRNA abundance |
| Pr20160 | ccccactatccgaacaacaa |  |
