# Supplementary material for: Streptococcus pneumoniae synchronizes the states of cell wall peptidoglycan acetylation and genome methylation by programmed DNA inversions
Source: PLoS Pathog. 2025 Aug 5;21(8):e1013286. doi: 10.1371/journal.ppat.1013286 (PMC12324116; doi:10.1371/journal.ppat.1013286)
Supplement: Attachment — Submitted filename: Responses to Reviewer 20250418.docx [file ppat.1013286.s021.docx]

**Responses to Reviewer’s Comments**

**Manuscript No:** PPATHOGENS-D-24-01692

### Title: *Streptococcus pneumoniae* synchronizes the states of cell wall peptidoglycan acetylation and genome methylation by programmed DNA inversions

**Date:** April 11, 2025

We greatly appreciate the reviewer’s insightful evaluation of our manuscripts. We have revised the entire paper according to the comments by performing additional experiments and modifying the text/figures. Thanks to reviewer’s valuable suggestions, the revised manuscript has been qualitatively improved from both the sufficiency of evidence and the depth of discussion. The revised manuscript has been submitted as requested, and the followings are my responses to the review comments.

**Part I – Summary**

**Reviewer #1:**

None

**Reviewer #2:**

None

**Reviewer #3:**

**In some ways, this is an outstanding study that presents some novel and highly interesting results. The presentation is generally clear and compelling. On the other hand, there are some issues about what** **state the bacteria are in physiologically and how the proposed mechanism could function. Notably, the study does not loop in other studies on** **the** **activation of LytA to the interpretation of the findings.**

This is indeed a very important aspect that we missed in our previous submission. We have thoroughly modified the Abstract, Introduction and Discussion sections, as well as the model figure (revised **Fig 9**) to make up this gap, with the citation of the previous studies in this area.

1. ****Line2 25, 375, 376, and throughout. The term *O*-de-acetylation is misleading and incorrect. It should be changed and throughout, especially in the Discussion. *O*-de-acetylation implies an enzymatic process, such as the GlcNAc deacetylation carried out by PdgA. No *O*-de-acetylase enzyme has been reported in pneumococcus. Please change “*O*-deacetylation” to “lack of *O-*acetylation,” “absence of *O-*acetylation” or “decreased *O-*acetylation”**

Revised.

2. **Lines 25-27.** **Typo (remove “of”). Also, please consider revising this sentence. It seems backward in that acetyl-Co-A is at the end of the sentence. Also, “physiological stages” is ambiguous.**

Revised.

3. **Line 49. Delete first “the”.**

Revised.

4. ****Indicate that O6 acetylation and attachment of WTAs are mutually exclusive. Please see comments later about LytA activation by WTA binding.**

Revised.

5. ****Line 67 after [16].** **Please rearrange Fig. 1 so that Fig. 1C is Fig. 1A, and include “(see Fig. 1A)” after “[16]”. It will be hard for readers to visualize this part without a diagram. Line 69. Ambiguous sentence. Consider changing to “and protects against autolysis”.**

Added (page 4, line 81). We have also rearranged **Fig 1**.

6. **Line 75. Typo.**

Revised.

7. ****Line 75. After [23,24] add “(see Fig. 2A)”. It will be hard for readers to visualize this part without a diagram.**

Revised. (page 5, line 105)

8. **Lines 91-95. Awkward sentence. Please rephrase.**

The last part of the Introduction has been rewritten. (page 5, lines 119-125)

9. ****Line 102.** **The type in all of the figures is much too small to read easily, especially the many bar diagrams. There is room to use larger size type in all of the figures and this should be done for clarity. Also, the reproduction of the larger figures was blurry and pixelated. Please these problems.**

We have modified all the figures as suggested.

10. ****Line 103 and everywhere.** **The term “****complementation” is not used properly in a genetic sense. Complementation means retaining a mutation in the chromosome and providing a separate wild-type copy of a gene ectopically. Complementation rules out polarity and other aberrations caused by the mutation and assigns function to the gene being interrogated. If I understand the strain table correctly, these “rev” strains contain repair of mutations back to WT. That is, the WT copy of the gene has replaced the mutant copy in the chromosome. The term “complementation” needs to be changed throughout. Were actual genetic complementation experiments performed? Some of the broader conclusions are weakened somewhat by lack of proper complementation experiments.**

Revised to “revertant”.

11. ****Line 103 and everywhere. The fact that point mutations were also constructed in most genes** **that were deleted and then repaired does tend to argue against polarity and downstream effects, although the way the strains are presented in the supplemental tables makes it somewhat difficult to tell where when antibiotic markers are present. Where these mutations markerless? In addition, point mutations often have the same phenotypes as the deletions, and it is not possible to tell without western blotting whether the mutants with amino acids were expressed or degraded. Was protein expression confirmed by western blotting in critical experiments?**

We have added gene modification information in figure legends to clarify the details. We performed Western blotting experiment to determine the expression of LytA in its deletion and point mutation strains. The data showed a comparable expression level of LytA in its point mutation strains with that of WT. Thus, the point mutation didn’t change the protein leve of Adr and LytA. The data are shown in **S3C Fig**.

12. ****Line 112. It is highly informative that similar results were detected in two other serotype strains. However, what happened in the serotype 2 strains in which most previous work on LytA and Adr was performed. This should be easy to check, because the authors have shown the rearrangements of hsdS in the D39 strain.**

We have tested *hsdS* inversions in strain D39 and its *adr* derivative. The data were presented in revised Results (pages 7-8, lines 192-196) and new **S2 Fig** showed significant impact of the PG NAM O-acetylation on the orientations of the *hsdS* genes.

13. **Line 128. Please consider changing “modulates” to “favors”.**

Revised.

14. **Line 162. Please consider changing “compensation” to “restoration” or “repair”.**

Revised.

15. ********Line 147. PgdA clearly modulates colony opacity, but not through the hsdS mechanism. Then PgdA is completely dropped from the study. How is PgdA doing this and does it impact the conclusions about Adr modifications? Please explain. Related to this question, why did A1 drop significantly in the pgdA mutant (Fig. 3B, column 2)**

We have discussed this issue in revised Results. (page 8, lines 214-219)

16. **Line 174. Remove “have” in sentence.**

Revised.

17. ****Line 196 and throughout. It has been shown that in cells untreated for antibiotics LytA is active primarily in stationary-phase cells. Cells in T/O colonies are likely in deep stationary phase. However, it is not clear in the other experiments what growth phase the cells were taken in. The one growth curve is in Figure 7D and the Methods suggest that cells were taken between 0.4 and 0.6 OD620, which appears to be early stationary phase. What happens in early to middle exponential phase? Does the epigenetic switching still occur when LytA is not active?**

We have addressed this issue by detecting *hsdS* inversion reactions at different phases of bacterial growth. The results shown in new **Figure 8F** and **8I** present that the proportion of *hsdS_A1_*-carrying bacteria reduced significantly at the early log phase when LytA-driven autolysis did not occur. This is consistent with our finding that the glycan-binding activity is essential for modulating *hsdS* inversions, but the other essential activities for autolysis (catalysis and choline-binding) are not necessary (see **Fig 4**). These lines of information argue that the regulatory function of LytA operates independent of its cell hydrolysis activity. We have added this point of view in the Discussion section. (page 17, lines 486-497)

18. ****Line 196, Fig. 7, and throughout. Along the same lines, some key papers about LytA function, such as** **Flores-Kim et al.** **eLife 8:e44912 2019 are not cited or considered in the model. Moreover, assuming that the model in Fig. 7F is correct, why wouldn’t the constitutive PG cell wall damage by LytA be lethal in the O-phase cells. Also, how accurate is the idea that colonizing pneumococci are starving during colonization (e.g., line 493)? Work by Samantha King and other others have shown that pneumococcal cells produce a variety of glycosyl transferases that cleave sugars off of the very abundant glycoproteins on epithelial cells. Please clarify this matter more.**

We’ve comprehensively revised the manuscript to include the functions LytA and WTA in the context of cell wall modifications and hydrolysis. (page 17, lines 486-497)

We’ve also added a statement in Discussion to elaborate on the nutrient availability issue. (pages 19-20, lines 584-588)

19. ****Line 222.** **Were western blots performed to determine whether these domains were actually expressed and at what relative levels?**

We have addressed this issue by Western blotting experiment using the anti-LytA polyclonal rabbit serum. The results were described in revised Results (page 9, line 261) and indicated that the expression of LytA in its deletion and point mutation variants under the *adr*^S438A^ background. While the active site point mutation variants *lytA*^E87A^, *lytA*^H133A^ and *lytA*^S33Q-Y41A^ showed comparable LytA expression with that of WT (**S3C Fig**), the domain deletion mutants Δ*lytA*^AMI^ and Δ*lytA*^CBD^ showed almost undetected LytA expression probably due to insufficient antigenic epitope to anti-LytA antibodies.

20. ****Line 248. The list in Table S2 contains some cytoplasmic proteins. Are you implying that these proteins bind to LytA before it is secreted? Are there any corroborative data (e.g., B2H assays) that PtvB binds to LytA?**

We believe that some of the proteins obtained from the LytA pulldown experiment are the non-specific contaminants, but it is possible that some of these proteins bind to LytA before being secreted. We have modified the text to reflect this point (page 10, lines 285-287).

We performed bacterial two-hybrid experiments to detect potential binding between the PtvB extracellular domain and LytA. The result in **S3E Fig** didn’t show a physical interaction of these two. We have added the data in the Results section. (page 11, lines 300-302).

21. ****Line 253. Is the involvement of PtvB in** **vancomycin tolerance dependent on LytA? This phenotype would support the model of an interaction.**

We addressed this issue by deleting *lytA* in various strain backgrounds and testing drug tolerance. Unfortunately, the loss of LytA itself made the wildtype bacteria more susceptible to vancomycin, which made it difficult to interpret the functional connection between LytA and PtvB. The data are shown in **S4 Fig**, and described in the text. (page 11, lines 303-306)

22. **Line 268. Awkward sentence; too many WTs. Please rephrase.**

Revised.

23. ****Line 279.** **As with the other proteomics data, the putative interactors seem to be all over the place. What does this mean?**

Some of the proteins might be pulled due to accidental fixation to LytA. We modified the text to reflect this possibility. (page 11, lines 324-326)

24. ****Line 287. Why is the methyltransferase mutant viable? Without methylation, why doesn’t the restriction enzyme kill the cell? Please explain.**

The restriction enzymes of the type-I restriction modification systems require the two other subunits to digest DNA: methyltransferase and sequence recognition subunits. Therefore, *hsdM* mutants are viable.

25. **Line 295.** **The dimA nomenclature is used in line 285, before naming it in line 295. Please move the naming up to around line 285.**

Revised.

26. **Line 303. Typo.**

Revised.

27. ****Line 308. This binding of DimA to PtvC seems marginal and not significant as stated. Did the reverse pair, show this binding more strongly? AS it stands, this is not a strong conclusion.**

We have constructed the reverse pair of the two proteins (T25-DimA and T18-PtvC) to detect potential interaction by BATCH assay. This experiment didn’t show any obvious interaction. The data are shown in **S5B Fig**.

28. **Line 316. Please include a protein interaction diagram to summarize the interactions and their relative strengths.**

We have included the protein interaction diagram for better understanding in the **Fig 6I**.

29. **Line 328 and Fig. 7B. Do you mean “rate” as stated in the figure or amount? Amount seems more appropriate.**

We have revised to “relative percentage” in both the main text and the figure legend.

30. ****Fig. 7D. The changes in the orientation of hsdS seem very modest. Are these very most changes sufficient to cause biological effects?**

We have traced the dynamic change of *hsdS_A1_* mRNA during a longer incubation period. Compared with almost 100% of *hsdS_A1_*-carrying bacteria at the exponential phase (3 h), the proportional abundance was steadily decreased from 58.3% at 4 h to 47.0% at 21 h (**Fig 8I**). Although to some extent, the T colonies proportion slightly but not significantly increased before 6.5 h (**Fig 8J**), the two-fold proportional alteration of *hsdS_A1_*-carrying bacteria in the population indeed resulted in different genome methylation patterns and biological effects.

31. ****Fig 7E.** **At what stage in growth are the glucose replete cultures. This growth curve does not seem correct. Is it pre-exponential? Please re-graph on a log scale.**

It is pre-exponential. To assure all group of the bacteria is in the similarly good condition, they were pre-cultured in C+Y medium until exponential phase, then washed and transferred into CDM with different concentration of glucose (see Method, page 21, line 607-610).

32. ****Line 351. Does the cellular amount of Adr protein change under the different growth conditions? Could a change in Adr protein amount contribute to these phenotypes?**

We have addressed this issue by detecting the mRNA of *adr* by qRT-PCR. The result showed that the expression of Adr in WT strain in CDM medium increased along with the higher glucose concentration, which was not correlated to the decline of intracellular Ac-CoA and *hsdS_A1_* proportion. The data are shown in **S6B Fig**.

33. **Line 373. Change “is” to “may”.**

Revised.

34. ****Line 385-401. Please see comments about LytA regulation and growth phase above. In addition, are any interactors of PsrA known that could tie together the results in this paper?**

We addressed this issue firstly by determining whether DimA modulates *hsdS* inversion by directly protein interact with PsrA. We assessed their potential interaction by BATCH assay. The result didn’t show any significant direct interaction between DimA and PsrA (new **Fig 7A**).

Since our recent study found that the up-regulation of recombinase PsrA gene led to the enrichment of T-phase allele and colonies, we compared the transcripts of T-dominant mutant *adr*^S438A^ with O-dominant WT and signal abolished mutant *adr*^S438A^*lytA*^S331-Y41A^ by RNA sequencing (**S5 and S6 Table** ). Compared with the parental WT strains, the *psrA* mRNA increased by 1.7 fold in *adr*^S438A^ mutant to favor the T colonies formation while declined by 5.5 fold in the signal abolished mutant *adr*^S438A^*lytA*^S331-Y41A^ (new **Fig 7**). This indicates that the LytA-mediated signal transduction of NAM O-acetylation status could control the transcriptional expression of recombinase PsrA gene to modulate *hsdS* inversion. Thus, we also detected the *psrA* mRNA in the *adr*^S438A^*∆dimA* mutant by qRT-PCR. Compared with the WT, the enriched *psrA* transcripts in *adr*^S438A^ was abolished by the knockout of *dimA* (new **Fig 7**). This suggests that DimA may transcriptionally regulation the recombinase PsrA to modulate the *hsdS* inversion under the low PG *O-*acetylation state. We arranged these data as an individual section of Results (pages 12-13, lines 361-396) and Figure (new **Fig 7**).

35. **Lines 402-409. The comparison to an anti-sigma factor mechanism is a bit confusing and could be left out.**

Revised.

36. ****Line 459. Are any of the proteins reported here included in the regulons of these TCSs? Please comment on this, because it could provide a link.**

The only link between the LytA-mediated regulation and the related studies is the impact of PsrA invertase gene upregulation (pages 12-13, lines 361-396). We’ve added a new **Fig 7** and a new section in Result.

37. **Line 482. Change “represents a major breakthrough” to “is a potential breakthrough” since the mechanism and growth phase dependence is far from clear.**

Revised.

38. **Line, 500. Add: “,respectively.”**

Revised.

39. ****Does O-acetylation significantly reduce WTA amount? Since TA binding is required for LytA activity, could this contribute to the block in LytA activity postulated in Fig. 7F right. Again, please provide context for the current interpretations based on previous functional studies of LytA, such as in Flores-Kim et al, 2019, listed above.**

Even though the *O*-acetylation and attachment of WTA on PG are mutually exclusive, the correlation between the ratio of these two decorations has never been reported. However, according to the finding of Flores-Kim et al., LytA-dependent pneumococcal autolysis is activated by the switching from LTA- to WTA-dominant TA synthesis, and the fact that amount of WTA in T-phase pneumococcal cells was much higher than that in O-phase cells (Kim and Weiser, J Infect Dis,1998), we hypothesized that the reduction of O-acetylation and non-HsdS_A1_ methylome state possibly accelerate the LTA-to-WTA switching, leading to the earlier and superior activation of LytA catalytic activity, which eventually cause T colony-dominant phenotype. We added this viewpoint in Discussion (page 17, lines 486-497) and modified the model in **Fig 9**.

40. ****As a general comment, as presented it is difficult to match the strains used in the experiments to the many bar graphs. The strain numbers from Table S4 should be listed in the figure legends.**

We have added the strain number into the colony morphology figures and figure legends to help better understand the key point.

**Part II – Major Issues**

**Reviewer #1:**

1. **The narrative implies that pneumococci have evolved to allow for survival during invasive infection. Since all forms of invasive infection are a dead end for this organism, this is difficult to accept as stated in the Introduction and Discussion sections. On the other hand, it seems more plausible that the organism adapts to different nutrient conditions, although these must be during colonization or transmission – the productive outcomes for the organism.**

We agreed with reviewer about this point. The phase switching between T and O is an adaptative reaction against diverse host niches. We have revised the description in both introduction and discussion.

2. **The important data in Figure 3 would be more convincing if a revertant of the key strain HsdSa1****adrS438A was included.**

We have addressed this issue by detecting the genome methylation of *adr*^rev^ strain by SMRT sequencing. The HsdS_A1_-specified methylation ratio (99.9%) has been successfully restored to that of WT level (98.3%) (**Fig 3C**) (page 8, lines 211-214).

3. **The transition to the consideration of LytA (and other choline binding proteins) is difficult to follow (beginning on lines 202-3). Please explain this in more detail. Of note, differences in LytA binding to O and T forms was previously reported (PMID:8675333) and should be discussed.**

We have explained this point with more details (pages 8-9, lines 227-232). Previous study has also demonstrated that the Adr rather than PgdA exerts an important role in resisting the lysis mediated by the major autolysin LytA (an amidase that an hydrolyzes cell wall PG). Nevertheless, there are other four known PG hydrolases (or autolysins) in *S. pneumoniae*, including LytB, LytC, CbpD, and PcsB, which recognize and cleave different bonds in pneumococcal cell wall. We thus tested the second possibility by mutagenesis analysis of LytA and other three genetically amendable PG hydrolases (LytB, LytC and CbpD), since Adr rather than PgdA has been demonstrated to exert an important role in LytA resistance.

4. **My major criticism is that differences in acetyl-CoA are described as an important aspect of regulation but never actually shown. Genes in the PDHC pathway (Fig. 7A) could have other effects and the mechanism described could be due to indirect consequences of manipulating this pathway. It would be more convincing to measure and directly manipulate acetyl-CoA levels to justify this key claim.**

We have confirmed the acetyl-CoA production in the genetic mutants of PDHC pathway. Consistent with the reported role for acetyl-CoA production, the deletion of *acoB* or *pfl* led to significant reduction of intracellular acetyl-CoA to 78.8% and 83% of WT level, respectively. The low PG *O-*acetylation mutant *adr*^S438A^ produced the lowest level of acetyl-CoA, which is only 59.3% of WT (**Fig 8B**). As compared with barely detectable PG *O-*acetylation in *adr*^S438A^ (1.5%), the relative percentage of the Mur*N*Ac acetylation was also significantly reduced in the Δ*acoB* (16.1%) and Δ*pfl* (21.5%) mutants compared with that of WT strain (35.7%) (**Fig 8C**). This result verified that the intracellular acetyl-CoA is utilized for the production of pneumococcal PG *O-*acetylation.

Then we assessed the growth curve of WT strain in TSB medium by detecting the optical density under 620 nm (OD_620_) and colony formatting unit (CFU). Both the OD and CFU values constantly increased and reached to a peak post 6.5-h incubation. The CFU dropped sharply after 6.5 h without a typical stationary phase while OD value kept stable until incubation for 21 h (**Fig 8F**). To trace the intracellular acetyl-CoA production during bacterial growth, we harvested the viable bacteria at the exponential (3 h post inoculation), early exponential (4 h post inoculation), middle exponential (5.5 h), late exponential (6.5 h), early death (8.5 h), and late death (21 h) phases (**Fig 8F**). Compared with the relative lower level of acetyl-CoA at the exponential phase, the whole exponential phase (4 h, 5.5 h, and 6.5 h) kept a 2-fold higher level of intracellular acetyl-CoA abundance. After entering the death phase, the intracellular acetyl-CoA concentration significantly declined to a quite low level (**Fig 8G**). Similar dynamic pattern was found in the rate of PG *O-*acetylation, which gradually increased from exponential to early-stationary phase while dropped to about a half after late-stationary phase (**Fig 8H**). This finding suggested that the amount of metabolite acetyl-CoA and PG *O-*acetylation changes along with nutrient’s density at different growth phase. To determine the impact of growth phase on *hsdS* inversions, we further quantified the relative abundance of *hsdS_A1_*-carrying bacteria and colony phenotype of bacteria collected from different incubation times. Compared with almost 100% of *hsdS_A1_*-carrying bacteria at the exponential phase (3 h), the proportional abundance was steadily decreased from 58.3% at 4 h to 47.0% at 21 h (**Fig 8I**).

We further verified this finding by growing pneumococci in a chemical defined medium (CDM) with various concentrations of glucose. Consistent with the glucose concentration-dependent growth (**Fig 8K**), there was a dose-dependent reduction both in the intracellular acetyl-CoA abundance (**Fig 8L**) and *hsdS_A1_*-carrying bacteria (**Fig 8M**) at 1.5 h post inoculation, at which the impact of glucose concentration on bacterial growth became obvious.

Taken together, the combined data strongly suggest that PG *O-*acetylation serves as an extracellular indicator of intracellular acetyl-CoA level to synchronize cellular carbon supply with bacterial metabolism, and finally modulate the epigenetic and colony phase switching.

5. **The final paragraph of the Results is not very convincing/impressive. Growth phase dependence only leads to a minor, albeit statistically significant, change in hsdSA1 abundance from 44.7 to 32.3% (Fig. 7D. Likewise the effect of glucose concentration (Fig. 7E) is small at best.**

We have traced the dynamic change of *hsdS_A1_* mRNA during a longer incubation period. Compared with almost 100% of *hsdS_A1_*-carrying bacteria at the exponential phase (3 h), the proportional abundance was steadily decreased from 58.3% at 4 h to 47.0% at 21 h (**Fig 8I**). Although to some extent, the T colonies proportion slightly but not significantly increased before 6.5 h (**Fig 8J**), the two-fold proportional alteration of *hsdS_A1_*-carrying bacteria in the population will indeed result in different genomic methylation patterns, which is sufficient to cause biological effects.

6. **Doesn’t deletion of lytA alone affect colony morphology? …..Independently of gene conversions? Can lytA mutants truly be compared (Fig4C) to lytA+?**

To determine whether the autolysin LytA can affect phase variation independent of the PG O-acetylation change, we constructed the *lytA* deletion mutant in WT background. The ∆*lytA* mutant produced 100% O colonies with no significant change of *hsdS_A1_* mRNA, which indicates that LytA modulate pneumococcal colony opacity in an *hsdS* inversion-independent manner (**S3A-B Fig**).

**Reviewer #2:**

**None**

**Reviewer #3:**

**See Part I**

**Part III – Minor Issues**

**Reviewer #1:**

1. **Line 43: surface rather than faces**

Revised.

2. **Line 105. The transition here is rough. Suggest changing to - Whole genome sequencing of hk11rev-N revealed……**

Revised.

3. **Line 119: Change to: replacement of serine at position 438 with alanine**

Revised.

4. **Line 132: Change The previous studies to: Previous studies**

Revised.

5. **Fig 7F.** **Please add a ? where the dashed lines are shown. As noted above, I would delete nasal colonization and invasive infection labels as this is not shown.**

Has been revised

**Reviewer #2:**

1. **Have the authors ever compared** **the % of PG O-acetylation of wild type:**

**a. In different growth phases or media?
b. With any of their mutants, e.g., lytA mutant?**

We have observed the percentage of PG O-acetylation of ST606 WT strain in different growth phases **(Fig 8H)**. The data shows the gradually increase of PG O-acetylation level from exponential to early-stationary phase and drastically reduced in late-stationary phase, which is almost synchronous with the change of intracellular acetyl-CoA.

2. **Similarly, have the authors measured intracellular actyl CoA in different growth phases or media and correlated with PG O-acetylation?**

See the response to major issue point 4 of Reviewer #1.

3. **Is LytA expression or activity altered depending on growth phases or media? Biofilm vs. non-biofilm growth? If so, there are several downstream predictions from the authors’ model that could be tested.**

Flores-Kim et al. has demonstrated that LytA-dependent pneumococcal autolysis is activated by the switching from LTA- to WTA-dominant TA synthesis. Another study also demonstrated that the amount of WTA in T-phase pneumococcal cells was much higher than that in O-phase cells (Kim and Weiser, J Infect Dis,1998). Therefore, we hypothesized that the reduction of *O-*acetylation and non-HsdS_A1_ methylome state possibly accelerate the LTA-to-WTA switching, leading to the earlier and superior activation of LytA catalytic activity, which eventually cause T colony-dominant phenotype. We added this viewpoint in Discussion (page 17, lines 486-497) and modified the model of **Fig 9**.

4. **Fig 1a. The naming of hkkrev-N and hkkrev strains is confusing. The “N” signifies the original strain that carries a mutation in the 5’ noncoding region of pdgA mutation, but the terminology seems non-intuitive, and the information is missing from the figure legend. Perhaps just an asterisk with note in legend that this indicates a spontaneous mutation in the non-coding region of pdgA.**

Revised as ∆*hk11*^rev*^.

5. **Line 34: In abstract, the meaning of "cross-membrane functional linkage" is unclear.**

Revised.

6. **Line 60. For clarity, I would mention "cell wall teichoic acids, which contain choline"**

Revised.

7. **Line 75. "determinant"**

Revised.

8. **Line 66. “subjected”**

Revised.

9. **A schematic figure illustrating *hsd* locus and inversion substrates would be useful.**

Revised by indicating a graphical representation (Fig 2A).

10. **Line 85.** **regulated by four of the 13 ...**

Revised.

11. **Line 86. Mutation of any of these four TCS led to ...**

Revised.

12. **Line 281. Upon first mention of dimA/myy1025, not that myy1025 is termed dimA based on evidence presented below.**

Revised.

13. **Line 282. Unclear sentence. Consider “****phenocopied the ptvB mutant in terms of …”**

Revised.

14. **Line 287. Not clear why the authors make this statement—explain further or delete.**

Deleted.

15. **Fig. 6H. The authors should comment on the much more subtle phenotype of the ptvC mutant compared to the ptvB mutant**

The only known function of PtvC is reported by our laboratory for its contribution to pneumococcal vancomycin tolerance together with PtvA and PtvB as an operon transcriptionally inhibited by the repressor PtvR (PMID: 28484041, already in the reference list). There is no any other reported function of PtvC independent of PtvB. We thus proposed there is possibly another protein as an alternative way to connection membrane-integrated PtvB and the intracellular effector DimA. This viewpoint has been added in the Discussion (page 17-18, line 514-515) and the proposed model in **Fig 6I and Fig 9**.

16. **Line 328. Suggest softening by replacing “required for” with “utilized in the production of”.**

Revised.

17. **Fig. 7 model and Line 394. A representation of the hsdS-A1-independent pathway to colony opacity should be added to the schematic. This might simply be some indication that this pathway is dependent on LytA catalytic activity, not simply PG binding, and perhaps an arrow with some question marks in the cytoplasmic space.**

We have added a model for clarifying the impact of LytA’s catalytic activity to the colony opacity in Fig. 7N.

18. **Line 444. The authors should also cite the extensive investigation into the role of Xis in directionality of lambda site-specific recombination (PMID: 25114241).**

We supplemented the discussion about Xis (page 18, lines 532-534).

19. **Line 489. The authors should consider “****via** **the extracellular regulatory circuit** **that involves PG O-acetylation”.**

Revised.

**Reviewer #3:**

See Part I
