# Supplementary material for: Streptococcus pneumoniae synchronizes the states of cell wall peptidoglycan acetylation and genome methylation by programmed DNA inversions
Source: PLoS Pathog. 2025 Aug 5;21(8):e1013286. doi: 10.1371/journal.ppat.1013286 (PMC12324116; doi:10.1371/journal.ppat.1013286)
Supplement: Attachment — Submitted filename: Response to Reviewers 2025-6-5.docx [file ppat.1013286.s022.docx]

**Response to Reviewers**

**Manuscript No:** PPATHOGENS-D-24-01692R1

### Title: *Streptococcus pneumoniae* synchronizes the states of cell wall peptidoglycan acetylation and genome methylation by programmed DNA inversions

**Date:** June 5, 2025

We greatly appreciate reviewers’ recognition of our manuscript and the suggestions proposed by review #1. We have revised the paper according to the comments by modifying the interpretation of our data in **Fig 8** and the related statement in the Abstract, Results and Discussion sections, as well as the model in **Fig 9**, which makes the revised manuscript more rigorous and logical.

**Part I – Summary**

**Reviewer #1: This is an excellent and thorough study of the relationship between cell wall modification and genome methylation. That said, this reviewer still finds the link to AcCoA levels and nutrient availability in Figure 8 to be greatly overstated in the revised manuscript.**

We fully agree with the viewpoint of reviewer #1 on the interpretation of our data in **Fig 8**, and have modified the related statement in the Abstract, Results, and Discussion sections (page 2, line 41-45; page 13, line 398; page 14, line 427-429; page 15, line 443-444; page 19, line 577-578), and **Fig 9** in the revised manuscript.

**Reviewer #2: Strong study made stronger by revision.**

**Reviewer #3: The authors did an excellent job in responding to all of the previous reviewer comments by adding data and revising parts of the manuscript. This is an interesting, important contribution to this area. The manuscript contains a large amount of high-quality data leading to solid conclusions and is presented well. I have no additional comments.**

**Part II – Major Issues: Key Experiments Required for Acceptance**

**Reviewer #1: While Figure 8 establishes an effect of growth phase on methylation, a direct relationship to AcCoA levels is still not demonstrated. For example, Fig. 8L shows a significant decrease in AcCoA levels between 2.5 and 1 g/L of glucose but the effect on hsdSa1 abundance in Fig. 8M is observed at a glucose concentration of between 20 and 10 g/L - a ten fold difference. This does not correlate. In my view, the claim in lines 41-45 (abstract) and corresponding section of the results and discussion (and model in Fig 9) about nutrient control needs to be substantially revised to remove any direct relationship to AcCoA.**

We fully agree this view on the interpretation of our data in **Fig 8** (the role of Ac-CoA in the regulatory circuit), and modified the Abstract, Results and Discussion sections in the revised manuscript (page 2, line 41-45; page 13, line 398; page 14, line 427-429; page 15, line 443-444; page 19, line 577-578). The overall model in **Fig 9** has also been modified to indicate a level of uncertainty, in terms of the role of acetyl-CoA in the regulatory circuit. The solid lines in the previous submission have been changed to dashed lines to connect the cell wall modification and cellular acetyl-CoA.

**Reviewer #2: None**

**Reviewer #3: None**

**Part III – Minor Issues**

**Reviewer #1:**

1. **The last 'summarizing' sentence in the abstract is confusing and should be simplified.**

Revised.

2. **The excessive use of strongly, dramatically,** **unequivocally, and striking are unnecessary and** **distract from the narrative. The strong data speaks for itself.**

Revised.

3. **line 122, overhauls? change word.**

Revised.

**Reviewer #2: None**

**Reviewer #3: None**
